# Targeting the bicarbonate transporter SLC4A4 overcomes immunosuppression and immunotherapy resistance in pancreatic cancer

**DOI:** 10.1038/s43018-022-00470-2

**Published:** 2022-12-15

**Authors:** Federica Cappellesso, Marie-Pauline Orban, Niranjan Shirgaonkar, Emanuele Berardi, Jens Serneels, Marie-Aline Neveu, Daria Di Molfetta, Francesca Piccapane, Rosa Caroppo, Lucantonio Debellis, Tessa Ostyn, Nicolas Joudiou, Lionel Mignion, Elena Richiardone, Bénédicte F. Jordan, Bernard Gallez, Cyril Corbet, Tania Roskams, Ramanuj DasGupta, Sabine Tejpar, Mario Di Matteo, Daniela Taverna, Stephan J. Reshkin, Baki Topal, Federico Virga, Massimiliano Mazzone

**Affiliations:** 1 Laboratory of Tumor Inflammation and Angiogenesis, Center for Cancer Biology, VIB, Leuven, Belgium; 2 Laboratory of Tumor Inflammation and Angiogenesis, Center for Cancer Biology, Department of Oncology, KU Leuven, Leuven, Belgium; 3 Laboratory of Precision Oncology and Cancer Evolution, Genome Institute of Singapore, A*STAR, Singapore, Singapore; 4 Department of Bioscience, Biotechnology and Biopharmaceutics, University of Bari, Bari, Italy; 5 Department of Imaging and Pathology, Translational Cell and Tissue Research, KU Leuven and University Hospitals Leuven, Leuven, Belgium; 6 Nuclear and Electron Spin Technologies Platform (NEST), Louvain Drug Research Institute, UCLouvain, Université Catholique de Louvain, Brussels, Belgium; 7 Biomedical Magnetic Resonance Research Group, Louvain Drug Research Institute, UCLouvain, Université Catholique de Louvain, Brussels, Belgium; 8 Pole of Pharmacology and Therapeutics (FATH), Institut de Recherche Expérmentale et Clinique (IREC), Université Catholique de Louvain, Brussels, Belgium; 9 Laboratory of Molecular Digestive Oncology, Department of Oncology, KU Leuven, Leuven, Belgium; 10 Department of Molecular Biotechnology and Health Sciences, University of Torino, Torino, Italy; 11 Molecular Biotechnology Center, University of Torino, Torino, Italy; 12 Department of Abdominal Surgery, University Hospitals Gasthuisberg Leuven and KU Leuven, Leuven, Belgium

**Keywords:** Immunotherapy, Cancer metabolism, Cancer, Pancreatic cancer

## Abstract

Solid tumors are generally characterized by an acidic tumor microenvironment (TME) that favors cancer progression, therapy resistance and immune evasion. By single-cell RNA-sequencing analysis in individuals with pancreatic ductal adenocarcinoma (PDAC), we reveal solute carrier family 4 member 4 (SLC4A4) as the most abundant bicarbonate transporter, predominantly expressed by epithelial ductal cells. Functionally, SLC4A4 inhibition in PDAC cancer cells mitigates the acidosis of the TME due to bicarbonate accumulation in the extracellular space and a decrease in lactate production by cancer cells as the result of reduced glycolysis. In PDAC-bearing mice, genetic or pharmacological SLC4A4 targeting improves T cell-mediated immune response and breaches macrophage-mediated immunosuppression, thus inhibiting tumor growth and metastases. In addition, *Slc4a4* targeting in combination with immune checkpoint blockade is able to overcome immunotherapy resistance and prolong survival. Overall, our data propose SLC4A4 as a therapeutic target to unleash an antitumor immune response in PDAC.

## Abstract (web summary)

Mazzone and colleagues identify the bicarbonate transporter SLC4A4 as highly abundant in epithelial duct cells in pancreatic cancer and show that its inhibition mitigates acidosis in the tumor, thereby alleviating immune suppression and overcoming immunotherapy resistance.

## Main

Tumors generally show a prominent decrease in the interstitial pH compared to healthy tissues, reaching values as low as 5.6 (ref. ^1^). Tumor acidity is emerging as a key driver of cancer progression because it can favor the selection of malignant cancer cells and, at the same time, can affect the composition and function of stromal cells present in the tumor microenvironment (TME)^1,2^.

In particular, acidity can blunt the antitumoral response of innate and adaptive tumor-infiltrating immune cells, thus contributing to immune escape^3–5^. Several studies have reported that, when exposed to high levels of lactate or to a low pH environment, T and natural killer cells become dysfunctional in favor of the expansion of immunosuppressive myeloid cells and regulatory T (T_reg_) cells^6–9^. In addition, tumor acidity can also directly affect the therapeutic efficacy of immune checkpoint inhibitors^5,10^. Therefore, targeting the main pH modulators to prevent tumor acidification is of fundamental importance in the context of antitumor immunity and immunotherapy. This is particularly true in the case of pancreatic ductal adenocarcinoma (PDAC), one of the most aggressive and lethal cancer types, where conventional therapies and the most recent immunotherapeutic approaches have failed to provide individuals with a promising treatment option^11^. This tumor type is characterized by a dense desmoplastic stroma that impedes oxygen and nutrient diffusion from the blood stream and contributes to a strong hypoxic and acidic TME^12^. Under physiological conditions, the pancreatic enzymes secreted by the acinar cells require an alkaline milieu for proper function; hence, bicarbonate is secreted by epithelial ductal cells against a sevenfold concentration gradient^13^. It follows that bicarbonate transporters could represent a valid target to tackle the pH aspect of the TME; however, their role remains largely unexplored.

Bicarbonate transporters are comprised of two families, SLC4 and SLC26, which can be further subdivided into acid loaders or acid extruders according to the directionality of the transport^14^. In particular, acid extruders absorb bicarbonate and may therefore be a suitable target to prevent the acidification of the TME. Within this group, there is *SLC4A4*, encoding an electrogenic sodium bicarbonate cotransporter (NBCe1) that, in normal tissues, is involved in pH regulation and homeostasis^15^. In humans, this transporter is present in three different splicing variants. One of the three variants (NBCe1-A) is expressed in the basolateral membrane of the renal proximal tubules where it mediates the transport of bicarbonate toward the blood. Another variant (NBCe1-B) is present in several organs but especially in the pancreatic ductal cells where its role is to accumulate bicarbonate in the intracellular space to allow the transfer of this metabolite from the blood to the lumen of the exocrine ducts^15^. Finally, a third variant (NBCe1-C) is exclusively expressed in the brain.

Contrasting evidence has proven that the reduction of SLC4A4 expression can both prohibit and promote cancer cell proliferation and migratory traits in vitro or in immunodeficient contexts, largely depending on the tumor cell type^16–20^. Nevertheless, to date, there are no in vivo studies that have examined SLC4A4 as a modulator of tumor pH and antitumor immune responses in pancreatic cancer. In this work, we explored the metabolic effects within the TME of *Slc4a4* deletion in cancer cells and the impact of these modifications on tumor growth, anticancer immunity and response to immunotherapy in mouse models of PDAC.

## Results

### *SLC4A4* is the most expressed bicarbonate transporter in PDAC

**Fig. 1:**
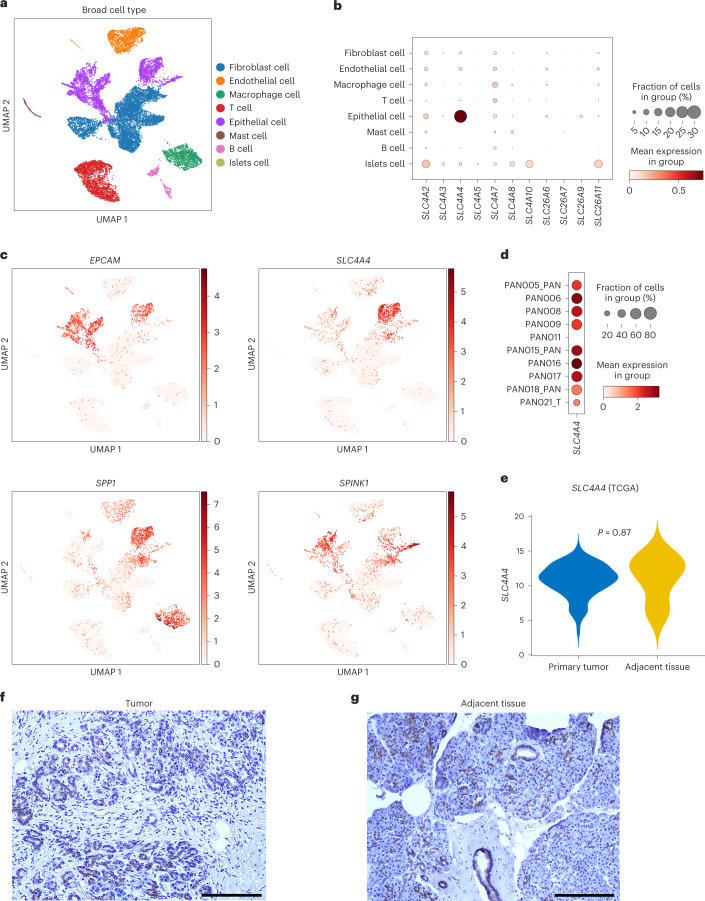
*SLC4A4* is almost exclusively expressed in the epithelial compartment of PDAC tumors. **a**, Uniform manifold approximation and projection (UMAP) map of color-coded cells for the indicated cell types isolated from treatment-naive individuals with PDAC (*n* = 10 individuals). **b**, Dot plot of normalized expression of different bicarbonate transporters in the indicated cell types. **c**, UMAPs showing the expression of *EPCAM* (marker of pan-epithelial cells), *SLC4A4*, *SPP1* (marker of ductal cells) and *SPINK1* (marker of acinar cells). **d**, Dot plot of normalized expression of *SLC4A4* in the epithelial compartment of each individual. **e**, Violin plot from TCGA data representing *SLC4A4* expression in PDAC or adjacent pancreatic tissue (*n* = 182 individuals). **f**,**g**, Representative images of IHC staining for SLC4A4 in PDAC (**f**) or adjacent pancreatic tissue sections (**g**); *n* = 7 individuals; scale bar, 1 mm. *P* value was assessed by unpaired, two-tailed Student’s *t*-test (**e**). Source data

**Extended Data Fig. 1:**
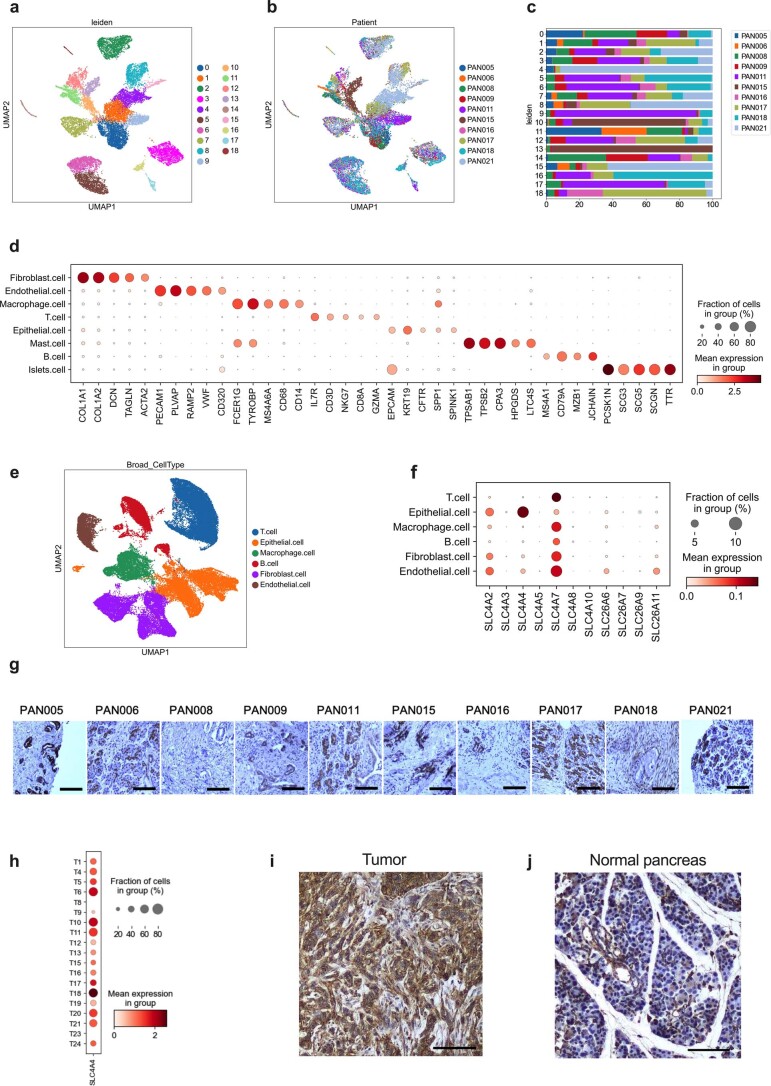
RNA-seq profiling and protein analysis for SLC4A4. **a**, Uniform Manifold Approximation and Projection (UMAP) representation of Leiden clusters identifying 19 cell types/states. **b**,**c**, UMAP (**b**) and proportionality bar plot (**c**) color-coded for individual patients. **d**, Dot plot showing the expression of marker genes used for cell type annotation. **e**, UMAP map of color-coded cells for the indicated cell types isolated from treatment-naïve PDAC patients (n = 24 patients). **f**, Dot plot of normalized expression of different bicarbonate transporters in the indicated cell types. **g**, Representative images of immunohistochemistry (IHC) staining for SLC4A4 in each patient from the RNA-seq cohort (n = 10 patients). **h**, Dot plot of normalized expression of *SLC4A4* in the epithelial compartment of each patient. **i**,**j**, Representative images of SLC4A4 IHC stainings in orthotopic KPC tumor sections (**i**) or normal pancreas (**j**) (n = 3 mice). Scale-bar: 0,5 mm (**g**,**i**,**j**). Source data

To identify clinically relevant candidates involved in the acidification of the TME, we analyzed the expression of bicarbonate transporters in single-cell RNA-sequencing (RNA-seq) data from a cohort of 10 treatment-naive individuals with PDAC. Leiden clustering and reference marker-based cell-type annotation revealed 19 distinct cell-state-specific clusters that are comprised of up to eight major cell types, including tumor epithelial cells, fibroblasts, T and B lymphocytes, macrophages and tumor endothelial cells, among others (Fig. 1a and Extended Data Fig. 1a–d). Among the bicarbonate transporters, we found *SLC4A4* to be most abundantly expressed in PDAC epithelial cells (Fig. 1b). Similar results were obtained by analyzing single-cell RNA-seq data from a separate cohort of 24 individuals with PDAC^21^ (Extended Data Fig. 1e,f). In particular, within the epithelial cluster (expressing epithelial cell adhesion molecule (*EPCAM*)), *SLC4A4* was mainly found in the ductal subcluster denoted by the expression of secreted phosphoprotein 1 (*SPP1*) but not in serine peptidase inhibitor kazal type 1 (*SPINK1*)-expressing acinar cells (Fig. 1c). SLC4A4 expression was prevalent in almost all individuals at the RNA (Fig. 1d and Extended Data Fig. 1h) and protein (Extended Data Fig. 1g) levels. Bulk RNA-seq comparison between PDAC and adjacent pancreatic tissue did not show any difference in *SLC4A4* expression (Fig. 1e). In accordance with the transcriptomic data, histological analysis showed that SLC4A4 expression was mostly restricted to the ductal cells within both the tumor and the adjacent pancreatic tissue (Fig. 1f,g). Similarly, staining for SLC4A4 in mice displayed a positive signal in the cancer cell compartment of orthotopic KPC tumors (obtained from the *Kras*^G12D^; *p53*^LSL.R172H^; *P48*:*Cre* transgenic mouse model) and in ducts and ductules of the surrounding pancreatic tissue (Extended Data Fig. 1i,j). The prevalent expression of SLC4A4 in tumor epithelial cells suggests that the inhibition of this transporter might predominantly affect cancer cells and have modest direct effects on other cell types of the TME.

### *Slc4a4* targeting inhibits tumor growth and metastasis

**Extended Data Fig. 2:**
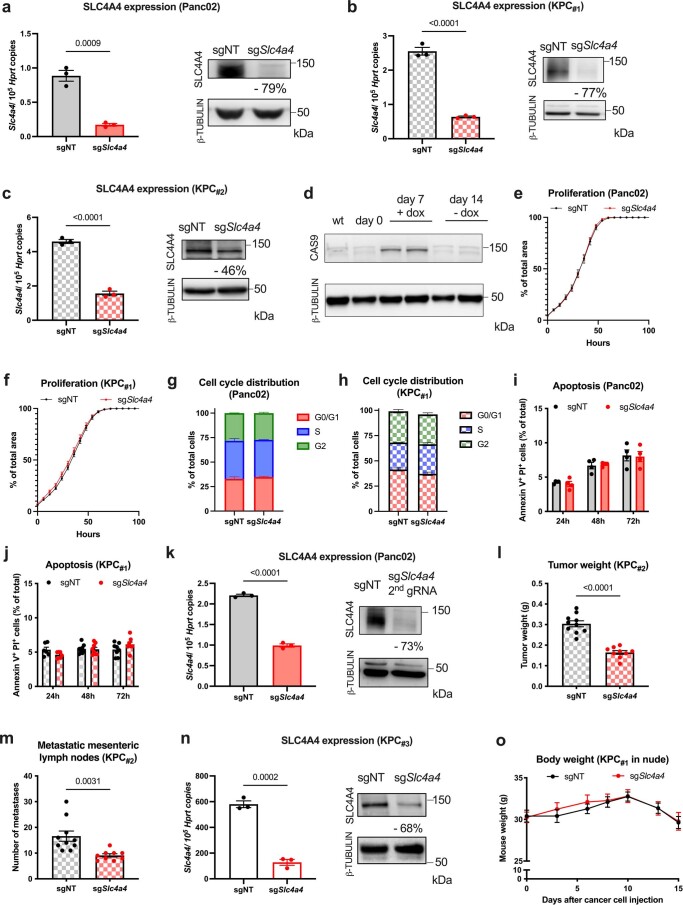
*Slc4a4* targeting does not affect *in vitro* proliferation, cell cycle and apoptosis of PDAC cells. **a**, SLC4A4 expression assessed by qRT-PCR (left, sgNT n = 3 and sg*Slc4a4* n = 3) and WB (right) analysis in Panc02 cells. **b**, SLC4A4 expression assessed by qRT-PCR (left, sgNT n = 3 and sg*Slc4a4* n = 3) and WB (right) analysis in KPC_#1_ cells. **c**, SLC4A4 expression assessed by qRT-PCR (left, sgNT n = 3 and sg*Slc4a4* n = 3) and WB (right) analysis in KPC_#2_ cells originating from a different clone than in **b**. **d**, CAS9 protein levels assessed by WB analysis before (day 0) or after 7 day of doxycycline treatment (day 7) followed by 7 day of doxycycline removal (day 14) in Panc02 cells. **e**,**f**, Proliferation curves assessed by Incucyte analysis of sgNT (n = 12) and sg*Slc4a4* (n = 12) Panc02 (**e**) or sgNT (n = 12) and sg*Slc4a4* (n = 12) KPC_#1_ (**f**) cells. **g**,**h**, Cell cycle distribution analysis (FACS) assessed by PI staining in sgNT (n = 4) and sg*Slc4a4* (n = 4) Panc02 (**g**) or sgNT (n = 12) and sg*Slc4a4* (n = 11) KPC_#1_ (**h**) cells. **i**,**j**, Percentage of PI^+^ Annexin V^+^ apoptotic cells (FACS) in sgNT (n = 4) and sg*Slc4a4* (n = 4) Panc02 (**i**) or in sgNT (n = 8) and sg*Slc4a4* (n = 8) KPC_#1_ (**j**) cells in culture for 24, 48 and 72 h. **k**, SLC4A4 expression assessed by qRT-PCR (left, sgNT n = 3 and sg*Slc4a4* (2^nd^ gRNA) n = 3) and WB (right) analysis in Panc02 cells. **l**,**m**, Weight (**l**) and quantification of macroscopic metastatic mesenteric lymph nodes (**m**) of sgNT (n = 10) and sg*Slc4a4* (n = 9) orthotopic KPC_#2_ tumors. **n**, SLC4A4 expression assessed by qRT-PCR (left, sgNT n = 3 and sg*Slc4a4* n = 3) and WB (right) analysis in KPC_#3_ cells **o**, Body weight of nude mice with sgNT (n = 8) and sg*Slc4a4* (n = 8) orthotopic KPC_#1_ tumors. The experiments in **a**,**b**,**c**,**d**,**k**,**n** have been repeated three times with similar results. n represents independently harvested cells (**a**-**c**,**e**-**k**,**n**). *P* value was assessed by unpaired Student’s t-test (**a**,**b**,**c**,**k**,**l**,**m**,**n**), and two-way ANOVA with Sidak’s multiple comparison test (**e**-**j**,**o**). Graphs show mean ± SEM. PI = propidium iodide. Source data

Based on the expression pattern of SLC4A4, we decided to study its functional relevance during PDAC progression in mice. First, we generated mouse Panc02 and KPC pancreatic cancer cells engineered with a doxycycline-inducible CRISPR–Cas9 system and either a single guide RNA (sgRNA) targeting *Slc4a4* (sgSlc4a4) or a non-targeting guide RNA (gRNA; sgNT) as a control (Extended Data Fig. 2a–c). All the experiments were performed after at least 1 week from doxycycline removal (Extended Data Fig. 2d). An important limitation of the Panc02 model is that these cells do not carry *Kras* mutations, which occur in 90% of human PDAC, but they are characterized by a loss-of-function mutation in the *Smad4* gene occurring in 50% of human pancreatic cancers^22^; moreover, this cell line is derived from a methylcholanthrene-induced adenocarcinoma, resulting in many more mutations and neoantigens than human PDAC; however, they retain resistance to immune checkpoint blockade (ICB)^23^. By contrast, KPC cells carry the most recurrent *Kras* and *Trp53* mutations found in individuals with PDAC and are overall poorly immunogenic^24^. Genetic *Slc4a4* targeting in both Panc02 and KPC cells did not affect cell proliferation, cell cycle distribution and apoptosis in vitro (Extended Data Fig. 2e–j).

**Fig. 2:**
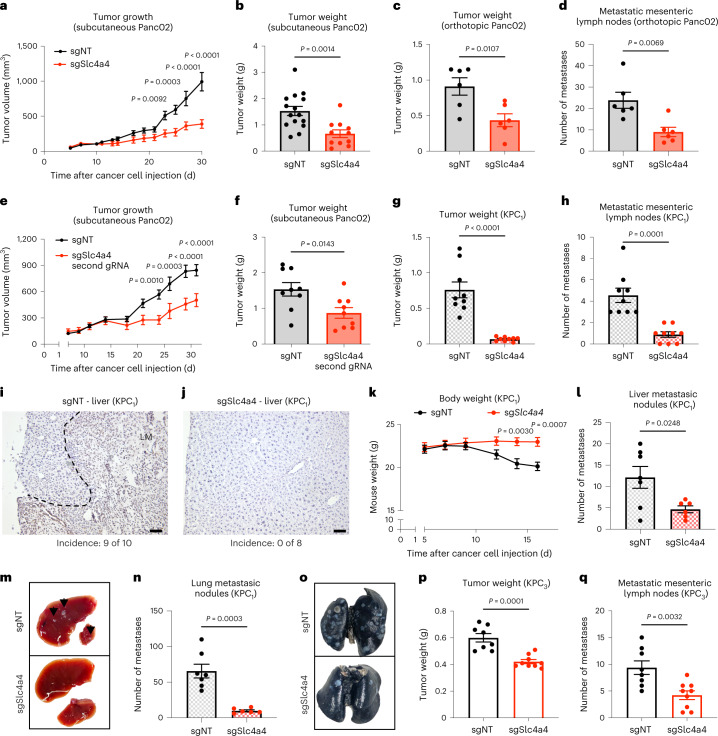
*Slc4a4* targeting reduces tumor growth and metastases. **a**, **b**, Growth (**a**) and weight (**b**) of sgNT (*n* = 15) and sgSlc4a4 (*n* = 11) subcutaneous Panc02 tumors. Data are representative of two independent experiments. **c**, Weight of sgNT (*n* = 6) and sgSlc4a4 (*n* = 6) orthotopic Panc02 tumors. Data are representative of two independent experiments. **d**, Quantification of macroscopic metastatic mesenteric lymph nodes of sgNT (*n* = 6) and sgSlc4a4 (*n* = 6) orthotopic Panc02 tumors. **e**, Tumor growth of sgNT (*n* = 9) and sgSlc4a4 (*n* = 8) subcutaneous Panc02 tumors using a second gRNA against *Slc4a4*. **f**, Weight of sgNT (*n* = 9) and sgSlc4a4 (*n* = 9) subcutaneous Panc02 tumors using a second gRNA against *Slc4a4*. **g**, Weight of sgNT (*n* = 9) and sgSlc4a4 (*n* = 9) orthotopic KPC_1_ tumors. Data are representative of two independent experiments. **h**, Quantification of macroscopic metastatic mesenteric lymph nodes of sgNT (*n* = 9) and sgSlc4a4 (*n* = 9) orthotopic KPC_1_ tumors. **i, j**, Representative images of CK19 in livers of sgNT (**i**; *n* = 10) and sgSlc4a4 (**j**; *n* = 8) orthotopic KPC_1_ tumor-bearing mice. The dashed line separates normal liver tissue from metastatic lesions. **k**, Body weight of sgNT (*n* = 9) and sgSlc4a4 (*n* = 9) orthotopic KPC_1_ tumor-bearing mice. **l**, **m**, Quantification (**l**) and representative images (**m**) of macroscopic liver metastatic nodules in mice hydrodynamically injected with sgNT (*n* = 7) and sgSlc4a4 (*n* = 6) KPC_1_ cells. Arrows indicate liver nodules (**m**). **n**, **o**, Quantification (**n**) and representative images (**o**) of macroscopic lung metastatic nodules in mice hydrodynamically injected with sgNT (*n* = 7) and sgSlc4a4 (*n* = 6) KPC_1_ cells. **p**, Weight of sgNT (*n* = 8) and sgSlc4a4 (*n* = 9) orthotopic KPC_3_ tumors. Data are representative of two independent experiments. **q**, Quantification of macroscopic metastatic mesenteric lymph nodes of sgNT (*n* = 8) and sgSlc4a4 (*n* = 9) orthotopic KPC_3_ tumors. *P* values were assessed by unpaired, two-tailed Student’s *t*-test (**b**–**d**, **f**–**h**, **l**, **n**, **p** and **q**) and two-way ANOVA with Sidak’s multiple comparison test (**a**, **e** and **k**). Graphs show mean ± s.e.m.; LM, liver metastasis; scale bars, 50 μm (**i** and **j**). Source data

Despite this, in subcutaneous Panc02 tumors, *Slc4a4* targeting strongly reduced tumor growth (Fig. 2a,b). Moreover, when Panc02 cancer cells were injected orthotopically into the pancreas, *Slc4a4* targeting led to a reduction of both tumor weight and number of metastatic mesenteric lymph nodes (Fig. 2c,d). We confirmed the same reduction in tumor growth with a second gRNA directed against *Slc4a4*, ruling out any (rare) off-target effect of the gRNA (Fig. 2e,f and Extended Data Fig. 2k).

These in vivo data were further validated using the clinically relevant KPC orthotopic tumors, which fully recapitulate the metabolic and histopathological features of human PDAC^25^. When engineered KPC cells (hereafter referred to as KPC_1_) were injected orthotopically in the pancreas, the effects of *Slc4a4* depletion were even more pronounced, with a reduction in tumor growth of almost 90% (Fig. 2g). The reduced aggressiveness of sgSlc4a4 tumors was associated with a marked decrease in the number of metastatic mesenteric lymph nodes (Fig. 2h) and in the number of hepatic metastases that were histologically detected in sgNT tumor-bearing mice (nine of ten mice and one to three lesions per liver cross-section) but not in sgSlc4a4 tumor-bearing mice (zero of eight mice; Fig. 2i,j). Remarkably, mice bearing *Slc4a4*-depleted KPC_1_ tumors did not show any body weight loss, while this was seen in control mice (Fig. 2k). In a second KPC clone (KPC_2_), despite a milder ablation of the targeted protein (Extended Data Fig. 2c), *Slc4a4* depletion led to an ~50% reduction in tumor growth and metastatic mesenteric lymph nodes (Extended Data Fig. 2l,m). To dissect the effect of *Slc4a4* depletion on metastatic growth independent from the effect on the primary tumor, we hydrodynamically injected KPC_1_ cells. In this setting, *Slc4a4* inactivation resulted in a strong decrease in metastatic colony formation both in the lung and in the liver (Fig. 2l–o). Moreover, we also inactivated *Slc4a4* in a third KPC clone (KPC_3_), which displayed increased aggressiveness, reduced T cell infiltration and higher resistance to immunotherapy (Extended Data Fig. 2n). Also with this ‘colder’ KPC clone, *Slc4a4* targeting led to a reduction in tumor growth and metastatic mesenteric lymph nodes (Fig. 2p,q).

**Fig. 3:**
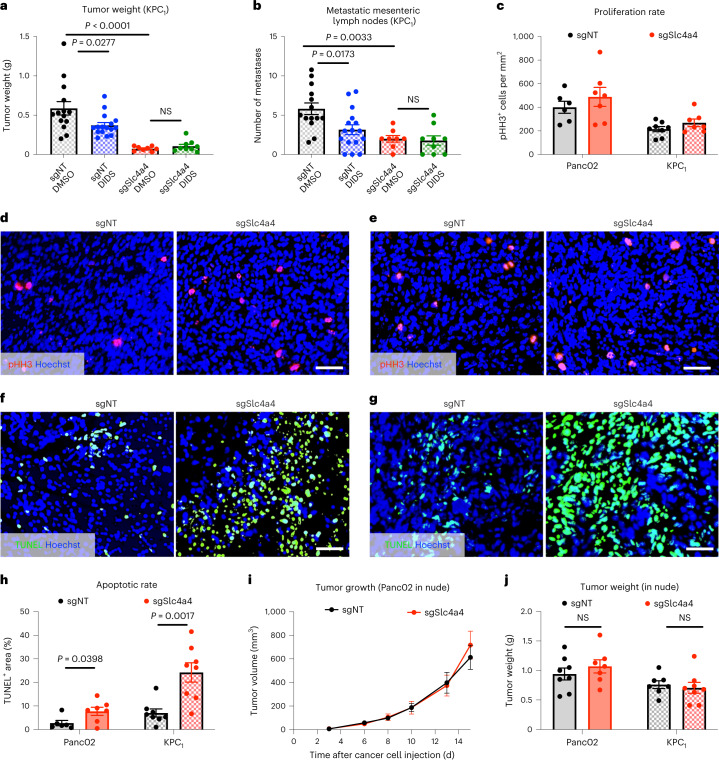
Genetic and pharmacologic *Slc4a4* targeting inhibits tumor growth and metastases in immunocompetent mice. **a**,**b**, Weight (**a**) and macroscopic metastatic mesenteric lymph nodes (**b**) of sgNT and sgSlc4a4 orthotopic KPC_1_ tumors in mice systemically treated with the SLC4A4 inhibitor DIDS (15 mg per kg (body weight) twice daily by i.p. injections) from day 5 to day 15 (sgNT DMSO *n* = 14, sgNT DIDS *n* = 16, sgSlc4a4 DMSO *n* = 8, sgSlc4a4 DIDS *n* = 9). Data show a pool of two independent experiments; NS, not significant. **c**,**d**, Quantification (**c**; left) and representative images (**d**) of pHH3 (red) in sgNT (*n* = 6) and sgSlc4a4 (*n* = 7) orthotopic Panc02 tumors. Hoechst (in blue) was used to stain the nuclei. **c**,**e**, Quantification (**c**; right) and representative images (**e**) of pHH3 (red) in sgNT (*n* = 8) and sgSlc4a4 (*n* = 7) orthotopic KPC_1_ tumors. Hoechst (in blue) was used to stain the nuclei. **f**,**h**, Representative images (**f**) and quantification (**h**; left) of TUNEL (green) stainings in sgNT (*n* = 6) and sgSlc4a4 (*n* = 7) orthotopic Panc02 tumors. Hoechst (in blue) was used to stain the nuclei. **g**,**h**, Representative images (**g**) and quantification (**h**; right) of TUNEL stainings (green) in sgNT (*n* = 8) and sgSlc4a4 (*n* = 8) orthotopic KPC_1_ tumors. Hoechst (in blue) was used to stain the nuclei. **i**,**j**, Growth (**i**) and weight (**j**; left) of sgNT (*n* = 8) and sgSlc4a4 (*n* = 8) subcutaneous Panc02 tumors in nude mice. **j**, Weight of sgNT (*n* = 7) and sgSlc4a4 (*n* = 8) KPC_1_ orthotopic tumors injected in nude mice (right). *P* values were assessed by two-way ANOVA with Tukey’s multiple comparison test (**a** and **b**), unpaired two-tailed Student’s *t*-test (**c**, **h** and **j**) and two-way ANOVA with Sidak’s multiple comparison test (**i**). Graphs show mean ± s.e.m.; scale bars, 20 μm (**d**–**g**). Source data

In addition to the genetic approach, we used the non-specific, commercially available SLC4A4 inhibitor 4,4′-diisothiocyano-2,2′-stilbenedisulfonic acid (DIDS) to pharmacologically and systemically target SLC4A4 (ref. ^26^). In this setting, mice bearing an orthotopic KPC_1_ tumor were treated twice daily with DIDS for 10 d starting on day 5 (from day 5 to day 15). Consistent with the genetic depletion, DIDS treatment led to a reduction in tumor growth and in the number of metastatic mesenteric lymph nodes in sgNT tumors. However, DIDS did not achieve a further reduction of sgSlc4a4 tumors and metastasis (Fig. 3a,b). These data suggest that, although this molecule is not a specific inhibitor of SLC4A4, the observed effect was greatly due to the inhibition of SLC4A4 rather than to general inhibition of bicarbonate transporters or other unrelated targets, pointing toward the therapeutic potential of systemic SLC4A4-targeted treatment.

Histological analysis of both Panc02 and KPC_1_ orthotopic tumors did not show any difference in terms of proliferation in sgSlc4a4 tumors compared to sgNT controls (Fig. 3c–e), whereas cell death was strongly augmented in both models following the targeting of *Slc4a4* (Fig. 3f–h). To discriminate cell-autonomous from non-cell-autonomous effects of SLC4A4 modulation in cancer cells, we orthotopically injected either Panc02 or KPC_1_ cells in immunodeficient nude mice. In this setting, sgNT and sgSlc4a4 tumors did not grow differently (Fig. 3i,j and Extended Data Fig. 2o). These data suggest the involvement of the immune system in the antitumor response observed after SLC4A4 depletion in cancer cells.

### *Slc4a4* targeting mitigates the acidification of the TME

**Fig. 4:**
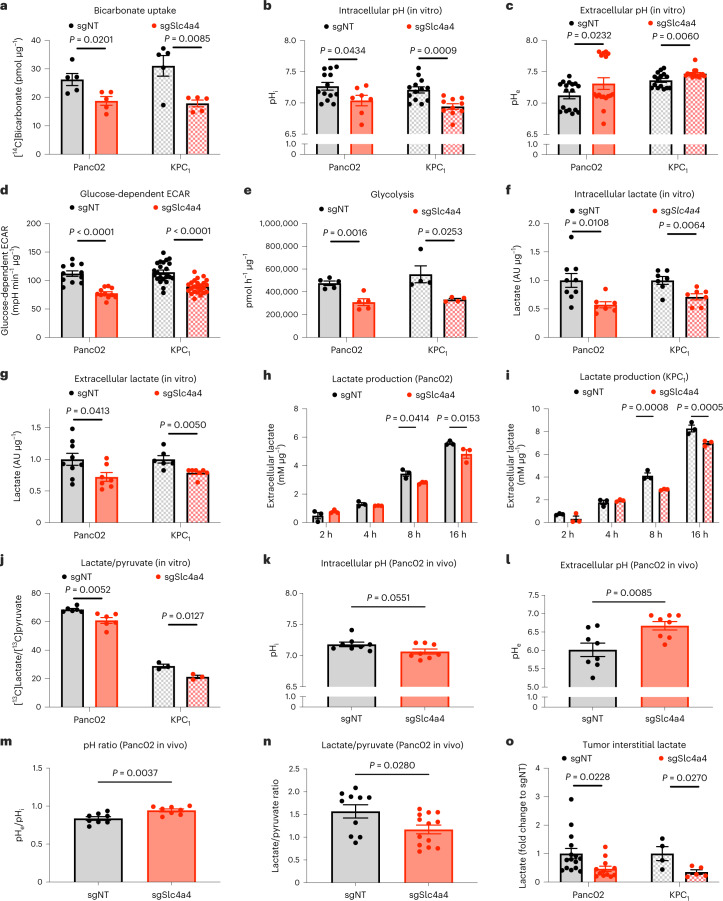
*Slc4a4* targeting decreases extracellular acidification and glycolysis. **a**, [^14^C]Bicarbonate uptake in sgNT (*n* = 5) and sgSlc4a4 (*n* = 5) Panc02 (left) and KPC_1_ (right) cells. **b**, pH_i_ in sgNT (*n* = 13 and 13) and sgSlc4a4 (*n* = 7 and 10) Panc02 (left) or KPC_1_ cells (right). **c**, pH_e_ in sgNT (*n* = 17 and 15) and sgSlc4a4 (*n* = 17 and 15) Panc02 (left) or KPC_1_ cells (right). **d**, Glucose-dependent ECAR in sgNT (*n* = 11 and 22) and sgSlc4a4 (*n* = 11 and 24) Panc02 (left) or KPC_1_ cells (right). **e**, ^3^H_2_O release from [^3^H]glucose in sgNT (*n* = 5 and 4) and sgSlc4a4 (*n* = 5 and 4) Panc02 (left) or KPC_1_ cells (right). **f**, Intracellular lactate levels measured by LC–MS in sgNT (*n* = 9 and 7) and sgSlc4a4 (*n* = 7 and 7) Panc02 (left) or KPC_1_ cells (right). **g**, Extracellular lactate levels measured by LC–MS in sgNT (*n* = 9 and 6) and sgSlc4a4 (*n* = 7 and 7) Panc02 (left) or KPC_1_ cells (right). **h**,**i**, Lactate measured by microdialysis in culture medium of sgNT (*n* = 3) and sgSlc4a4 (*n* = 3) Panc02 (**h**) or KPC_1_ (**i**) cells at 2, 4, 8 and 16 h. **j**, [^13^C]Lactate to [^13^C]pyruvate ratio in sgNT (*n* = 6 and 3) and sgSlc4a4 (*n* = 6 and 3) Panc02 (left) or KPC_1_ cells (right). **k**–**m**, pH_i_ (**k**), pH_e_ (**l**) and pH ratio (**m**) in sgNT (*n* = 8) and sgSlc4a4 (*n* = 8) subcutaneous Panc02 tumors assessed by ^31^P-MRS. Data show a pool of two independent experiments. **n**, Lactate to pyruvate ratio calculated from area under the curve (AUC) in sgNT (*n* = 10) and sgSlc4a4 (*n* = 13) subcutaneous Panc02 tumors assessed by MRS following the administration of hyperpolarized pyruvate. Data show a pool of three independent experiments. **o**, Lactate concentration measured by LC–MS in tumor interstitial fluid of sgNT (*n* = 15) and sgSlc4a4 (*n* = 12) Panc02 subcutaneous tumors (left) or of sgNT (*n* = 4) and sgSlc4a4 (*n* = 5) orthotopic KPC_1_ tumors (right). Data show a pool of two independent experiments (left). Data were normalized by protein content (**a** and **d**–**i**); *n* represents independently collected cells (**a**–**j**). *P* values were assessed by unpaired two-tailed Student’s *t*-test (**a**, **b**, **d**–**g** and **j**–**o**), paired two-tailed Student’s *t*-test (**c**) and two-way ANOVA with Sidak’s multiple comparison test (**h** and **i**). Graphs show mean ± s.e.m.; AU, arbitrary units. Source data

In light of the role of SLC4A4 as a bicarbonate importer in the pancreas^27^, *Slc4a4* targeting resulted in a substantial reduction of bicarbonate uptake in both Panc02 and KPC_1_ cells (Fig. 4a). Given the importance of the bicarbonate buffer system for pH homeostasis^15^, we performed an extensive analysis of the pH dynamics in vitro. Here, we found that targeting *Slc4a4* in Panc02 cells led to a decrease in the intracellular pH (pH_i_) along with an increase of the extracellular pH (pH_e_; Fig. 4b,c, left). Similar modulations of the pH_i_ and pH_e_ after *Slc4a4* depletion were obtained in the KPC_1_ cell line (Fig. 4b,c, right). Overall, these data further corroborate an extracellular accumulation of bicarbonate in sgSlc4a4 cells, acting as a buffer in the milieu.

**Extended Data Fig. 3:**
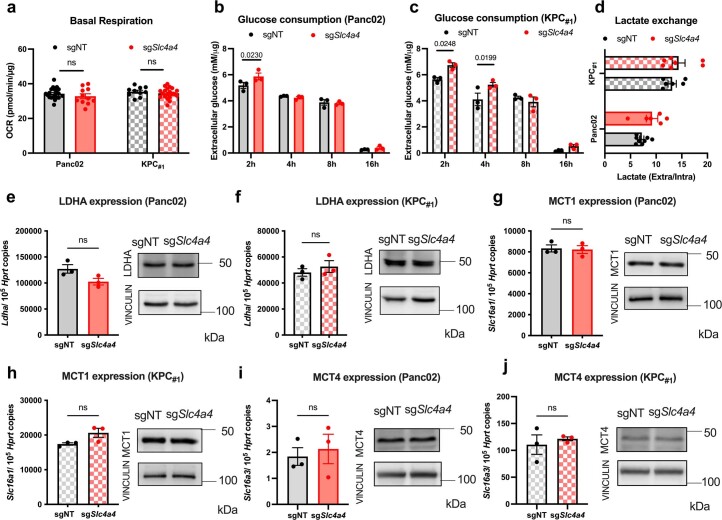
*Slc4a4* targeting does not affect LDHA, MCT1, MCT4 expression. **a**, Basal OCR in sgNT (n = 20) and sg*Slc4a4* (n = 11) Panc02 and sgNT (n = 10) and sg*Slc4a4* (n = 20) KPC_#1_ cells. **b**, Glucose measured by microdialysis in culture medium of sgNT (n = 3) and sg*Slc4a4* (n = 3) Panc02 cells at 2, 4, 8 and 16 h. Data are normalized by protein content. **c**, Glucose measured by microdialysis in culture medium of sgNT (n = 3) and sg*Slc4a4* (n = 3) KPC_#1_ cells at 2, 4, 8 and 16 h. Data are normalized by protein content. **d**, Extracellular to intracellular lactate ratio measured by LC/MS in sgNT (n = 8) and sg*Slc4a4* (n = 6) Panc02 cells (bottom) and in sgNT (n = 6) and sg*Slc4a4* (n = 7) KPC#1 cells (top). **e**, LDHA expression assessed by qRT-PCR (left, sgNT n = 3 and sg*Slc4a4* n = 3) and WB (right) analysis in Panc02 cells. **f**, LDHA expression assessed by qRT-PCR (left, sgNT n = 3 and sg*Slc4a4* n = 3) and WB (right) analysis in KPC_#1_ cells. **g**, MCT1 expression assessed by qRT-PCR (left, sgNT n = 3 and sg*Slc4a4* n = 3) and WB (right) analysis in Panc02 cells. **h**, MCT1 expression assessed by qRT-PCR (left, sgNT n = 3 and sg*Slc4a4* n = 3) and WB (right) analysis in KPC_#1_ cells. **i**, MCT4 expression assessed by qRT-PCR (left, sgNT n = 3 and sg*Slc4a4* n = 3) and WB (right) analysis in Panc02 cells. **j**, MCT4 expression assessed by qRT-PCR (left, sgNT n = 3 and sg*Slc4a4* n = 3) and WB (right) analysis in KPC_#1_ cells. The experiments in **e**-**j** have been repeated three times with similar results. n represents independently harvested cells. *P* value was assessed by unpaired, two-tailed Student’s t-test (**a**,**d**-**j**) and two-way ANOVA with Sidak’s multiple comparison test (**b**-**c**).Graphs show mean ± SEM. Source data

A small difference in the pH_i_ can cause radical metabolic changes^28,29^. Seahorse analysis revealed that *Slc4a4* targeting impaired the glycolytic rate in both Panc02 and KPC_1_ cells as glucose-dependent extracellular acidification rate (ECAR) was reduced (Fig. 4d) but did not affect the basal oxygen consumption rate (OCR; Extended Data Fig. 3a). Further experiments with [^3^H]glucose confirmed a lower glycolytic flux in sgSlc4a4 cells than in sgNT control cells in both models (Fig. 4e), which was corroborated by decreased levels of both intracellular and extracellular lactate, as measured by liquid chromatography–mass spectrometry (LC–MS; Fig. 4f,g). Consistently, compared to sgNT, the culture medium of sgSlc4a4 cells displayed a gradual decrease in lactate accumulation over time (Fig. 4h,i) and a reduction in glucose consumption only at early time points (Extended Data Fig. 3b,c). Reduced glycolysis and lactate production in sgSlc4a4 cancer cells likely reflects the inhibitory effect of a lower pH_i_ on the activity of glycolytic enzymes including hexokinase, 6-phosphofructokinase and lactate dehydrogenase A (LDHA), the latter converting pyruvate into lactate^28,30–32^. This inhibitory effect of acidity on glycolytic enzymes represents a negative feedback loop to protect the cell from suffering massive intracellular acidification as glycolysis represents a proton source^33,34^. In fact, LDHA activity measured with [^13^C]pyruvate tracing through LC–MS showed a reduction of ^13^C incorporation into lactate in both Panc02 and KPC_1_ sgSlc4a4 cells compared to sgNT controls (Fig. 4j). The ratio between extracellular and intracellular lactate did not change (Extended Data Fig. 3d). Moreover, RNA and protein levels of LDHA and of the monocarboxylate transporter 1 and 4 (MCT1 and MCT4) were comparable in sgNT and sgSlc4a4 Panc02 or KPC_1_ cells (Extended Data Fig. 3e–j), altogether arguing that the lactate exchanging machinery was not altered. All these data indicate that besides the direct impairment in bicarbonate absorption, SLC4A4 inhibition indirectly drives metabolic changes that further decrease extracellular acidity through a reduction in lactate production.

**Extended Data Fig. 4:**
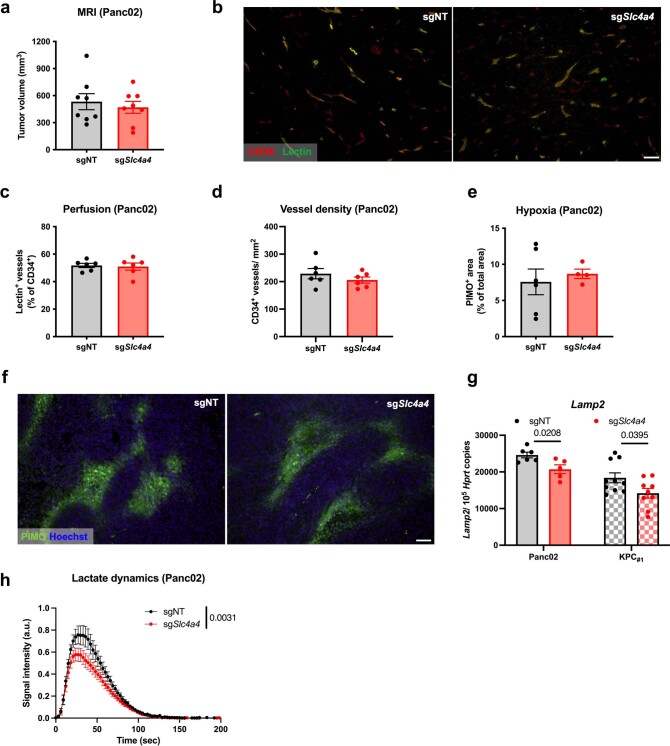
*Slc4a4* targeting does not alter tumor perfusion and hypoxia. **a**, Size of sgNT (n = 8) and sg*Slc4a4* (n = 8) subcutaneous Panc02 tumors assessed by MRI. Data show a pool of two independent experiments. **b**, Representative images of lectin^+^ (in green) perfused vessels (in red, CD34^+^) in sgNT (n = 6) and sg*Slc4a4* (n = 6) subcutaneous Panc02 tumors. **c**, Quantification of vessel perfusion in sgNT (n = 6) and sg*Slc4a4* (n = 6) subcutaneous Panc02 tumors. **d**, Quantification of vessel density in sgNT (n = 6) and sg*Slc4a4* (n = 6) subcutaneous Panc02 tumors. **e**,**f**, Quantification (**e**) and representative images (**f**) of hypoxic areas (in green, PIMO^+^) in sgNT (n = 6) and sg*Slc4a4* (n = 4) subcutaneous Panc02 tumors. Hoechst (in blue) was used to stain the nuclei. **g**, *Lamp2* expression assessed by qRT-PCR analysis in sgNT (n = 6) and sg*Slc4a4* (n = 5) subcutaneous Panc02 tumors (left) or in sgNT (n = 10) and sg*Slc4a4* (n = 9) orthotopic KPC_#1_ tumors (right). **h**, Dynamics of lactate production in sgNT (n = 10) and sg*Slc4a4* (n = 13) subcutaneous Panc02 tumors assessed by MRS following the administration of hyperpolarized pyruvate. Data show a pool of three independent experiments. *P* value was assessed by unpaired, two-tailed Student’s t-test (**a**,**c**-**e**,**g**,**h**). Graphs show mean ± SEM. Scale bars: 50 μm (**b**,**f**). a.u., arbitrary unit. Source data

To confirm the observed metabolic alterations in vivo, we measured both pH_i_ and pH_e_ by the aid of ^31^P-magnetic resonance spectroscopy (MRS). In size-matched tumors (when the volumes of sgNT and sgSlc4a4 tumors were still comparable; Extended Data Fig. 4a), we determined that in sgSlc4a4 tumors, the intracellular environment was more acidic and the extracellular space was more alkaline than in sgNT tumors (Fig. 4k–m). No differences in tumor vessel perfusion, density or hypoxia were observed, making technical issues related to probe diffusion within the tumor unlikely (Extended Data Fig. 4b–f). Given the evidence that lysosome-associated membrane protein 2 (LAMP2) relocalization and expression induction have been associated with the acidification of the TME^35^, we analyzed *Lamp2* levels in total RNA lysates from tumors. Expression of *Lamp2* was decreased in both Panc02 and KPC_1_ sgSlc4a4 tumors compared to the sgNT controls (Extended Data Fig. 4g), supporting our conclusion that *Slc4a4* depletion in cancer cells is sufficient to mitigate the acidic pH of the TME in PDAC.

To further support the idea that decreased lactate concentration in the extracellular space is a consequence of diminished LDHA activity in response to a lower pH_i_^28^ and on the basis of the differences in lactate levels observed in vitro (Fig. 4f–i), we used MRS to assess in real time the transformation of hyperpolarized [^13^C]pyruvate into lactate, which mainly reflects LDHA activity^36^. Because this method is technically challenging on visceral tissues/tumors, we used subcutaneous Panc02 tumors where we observed a decrease in the lactate to pyruvate ratio (Fig. 4n and Extended Data Fig. 4h). Finally, to support the idea that this reduction in LDHA activity could result in lower extracellular lactate accumulation, we analyzed the interstitial fluid of both subcutaneous Panc02 and orthotopic KPC_1_ tumors where lactate levels were decreased in the case of *Slc4a4* targeting (Fig. 4o).

### *Slc4a4* targeting reinvigorates CD8^+^ T cell response

**Fig. 5:**
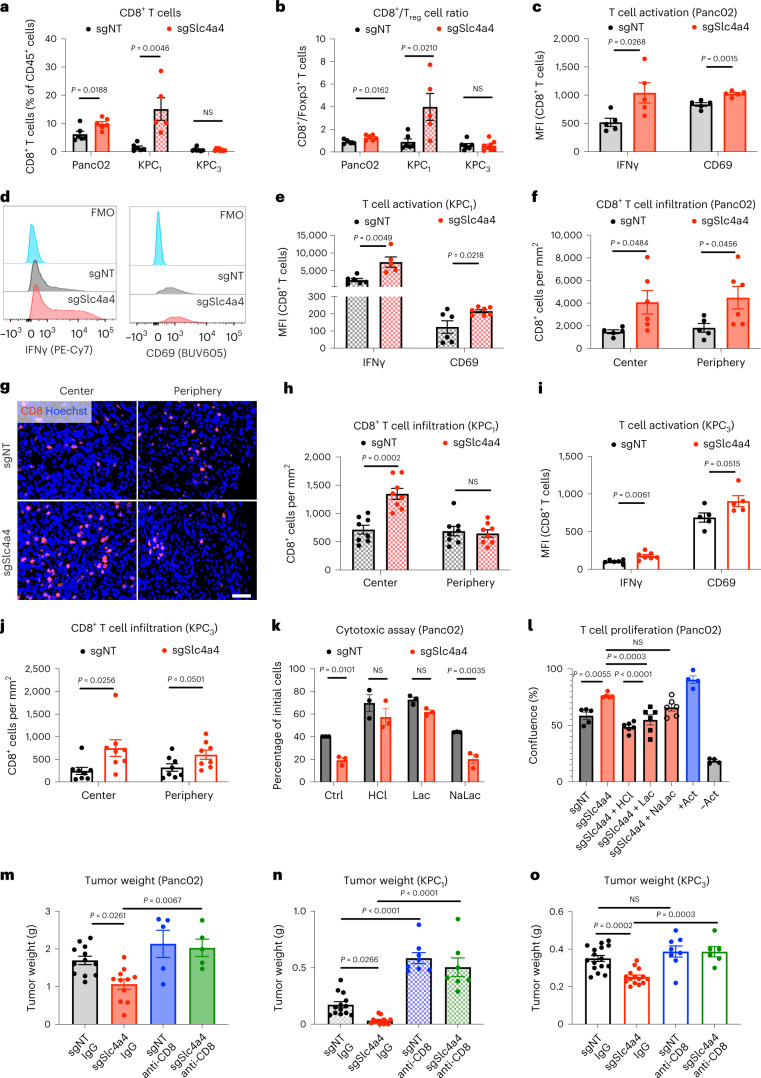
*Slc4a4* targeting unleashes a CD8^+^ T cell-mediated immune response. **a**, Percentage of CD8^+^ cells in sgNT (*n* = 6, 6 and 7) or sgSlc4a4 (*n* = 6, 5 and 8) subcutaneous Panc02 (left), orthotopic KPC_1_ (middle) and KPC_3_ tumors (right). **b**, CD8^+^:T_reg_ cell ratio in sgNT (*n* = 5, 6 and 7) and sgSlc4a4 (*n* = 6, 5 and 8) subcutaneous Panc02 (left), orthotopic KPC_1_ (middle) and KPC_3_ tumors (right). **c**,**d**, Quantification (**c**) and representative histograms (**d**) of the MFI of IFNγ (left) and CD69 (right) in CD8^+^ cells in sgNT (*n* = 5) and sgSlc4a4 (*n* = 5) subcutaneous Panc02 tumors. FMO: fluorescence minus one. **e**, MFI of IFNγ (left) and CD69 (right) in CD8^+^ cells in sgNT (*n* = 6 and 6) and sgSlc4a4 (*n* = 5 and 7) orthotopic KPC_1_ tumors. **f**, CD8 staining in sgNT (*n* = 5) and sgSlc4a4 (*n* = 6) orthotopic Panc02 tumors. **g**,**h**, Representative images (**g**) and quantification (**h**) of CD8 (red) staining in sgNT (*n* = 8) and sgSlc4a4 (*n* = 8) orthotopic KPC_1_ tumors. Nuclei are stained with Hoechst (blue). **i**, MFI of IFNγ (left) and CD69 (right) in CD8^+^ cells in sgNT (*n* = 6 and 5) and sgSlc4a4 (*n* = 7 and 5) orthotopic KPC_3_ tumors. **j**, CD8 staining in orthotopic sgNT (*n* = 8) and sgSlc4a4 (*n* = 8) KPC_3_ tumors. **k**, Viable (%) Panc02-ovalbumin (Panc02-OVA) cells cocultured with OT-1 T cells in T cell medium (control (Ctrl), *n* = 3) with lactic acid (Lac; *n* = 3), HCl (*n* = 3) or sodium lactate (NaLac; *n* = 3). **l**, CD8^+^ T cell proliferation cultured in conditioned medium from sgNT (*n* = 5) or sgSlc4a4 (*n* = 5) Panc02 cells supplemented with Lac (*n* = 6), HCl (*n* = 6) or NaLac (*n* = 6); +act, maximal; –act, basal. **m**, Weight of subcutaneous Panc02 tumors in mice treated with anti-CD8 or control IgG (IgG; sgNT-IgG *n* = 12, sgSlc4a4-IgG *n* = 11, sgNT-anti-CD8 *n* = 5 and sgSlc4a4-anti-CD8 *n* = 5). **n**, Weight of orthotopic KPC_1_ tumors in mice treated with anti-CD8 or IgG (sgNT-IgG *n* = 13, sgSlc4a4-IgG *n* = 13, sgNT-anti-CD8 *n* = 8 and sgSlc4a4-anti-CD8 *n* = 7). **o**, Weight of orthotopic KPC_3_ tumors in mice treated with anti-CD8 or IgG (sgNT-IgG *n* = 15, sgSlc4a4-IgG *n* = 14, sgNT-anti-CD8 *n* = 7 and sgSlc4a4-anti-CD8 *n* = 5). Data in **m**–**o** are representative of pools of two independent experiments. *n* represents independently collected cells (**k** and **l**). *P* value was assessed by unpaired, two-tailed Student’s *t*-test (**a**–**c**, **e**, **f** and **h**–**j**), two-way ANOVA with Sidak’s multiple comparison test (**k**), one-way ANOVA with Tukey’s multiple comparison test (**l**) and two-way ANOVA with Tukey’s multiple comparison test (**m**–**o**). Graphs show mean ± s.e.m.; scale bar, 20 μm (**g**). Source data

**Extended Data Fig. 5:**
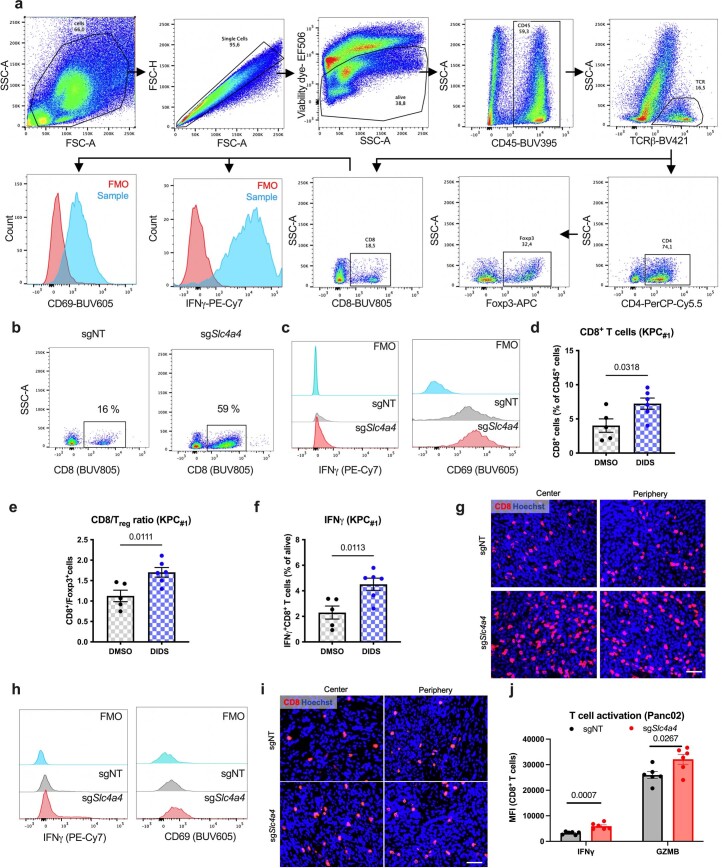
Central role of CD8^+^ T cells in the anti-tumor phenotype following the targeting of *Slc4a4* in cancer cells. **a**, Gating strategy for T cell compartment (numbers represent percentage of cells out of parental population). **b**, Representative flow cytometry plots of CD8^+^ T cell within sgNT and sg*Slc4a4* tumors. **c**, Representative histograms of the MFI of IFNγ (left) and CD69 (right) in CD8^+^ T cells (FACS) in sgNT and sg*Slc4a4* orthotopic KPC_#1_ tumors. **d**, Percentage of CD8^+^ T cells (FACS) in KPC_#1_ orthotopic tumors in DMSO- (n = 5) or DIDS- (n = 6) treated mice. **e**, CD8^+^ to T_reg_ cell ratio obtained by dividing the percentage of CD8^+^ T cells (out of CD45^+^ cells) by the percentage of Foxp3^+^CD4^+^ T cells (out of CD45^+^ cells) in orthotopic KPC_#1_ tumors from DMSO- (n = 5) or DIDS- (n = 6) treated mice. **f**, Percentage of IFNγ in CD8^+^ T cells (FACS) in orthotopic KPC_#1_ tumors from DMSO- (n = 5) or DIDS- (n = 6) treated mice. **g**, Representative pictures of CD8 (in red) immunofluorescence stainings in sgNT (n = 5) and sg*Slc4a4* (n = 6) orthotopic Panc02 tumors. Hoechst (in blue) was used to stain the nuclei. **h**, Representative histograms of the MFI of IFNγ (left) and CD69 (right) in CD8^+^ T cells (FACS) in orthotopic KPC_#3_ tumors. **i**, Representative pictures of CD8 (in red) immunofluorescence stainings in sgNT (n = 8) and sg*Slc4a4* (n = 8) orthotopic KPC_#3_ tumors. Hoechst (in blue) was used to stain the nuclei. **j**, MFI of IFNγ (left) and GZMB (right) in CD8^+^ OT-I T cells (FACS) after 24-hour co-culture with sgNT (n = 6) and sg*Slc4a4* (n = 6) Panc02-OVA cells. n represents independently harvested cells (**j**). *P* value was assessed by unpaired, two-tailed Student’s t-test (**d**,**e**,**f**,**j**). Graphs show mean ± SEM. Scale bars: 20 μm (**g**,**i**). Source data

Based on the well-documented interplay between pH and immune response^6–9^, and the absence of an antitumor effect of *Slc4a4* inhibition in immunodeficient mice, we studied the immune landscape after SLC4A4 depletion in cancer cells. Flow cytometry analysis (Extended Data Fig. 5a) revealed that sgSlc4a4 Panc02 tumors displayed an increase in CD8^+^ T cell infiltration and CD8^+^/T_reg_ cell ratio (Fig. 5a,b, left, and Extended Data Fig. 5b), with augmented expression of the activation marker CD69 and increased secretion of the effector cytokine IFNγ (Fig. 5c,d). The same immune phenotype was confirmed in the orthotopic KPC_1_ model, where *Slc4a4* targeting resulted in a tenfold higher CD8^+^ T cell accumulation (Fig. 5a, middle) and increased CD8^+^/T_reg_ cell ratio (Fig. 5b, middle), CD69 expression and IFNγ production (Fig. 5e and Extended Data Fig. 5c). Similarly, flow cytometric analysis of the immune infiltrate of tumors treated with the SLC4A4 inhibitor DIDS recapitulated the immune phenotype induced by genetic deletion of *Slc4a4*, with augmented CD8^+^ T cell infiltration, CD8^+^/T_reg_ cell ratio and IFNγ expression (Extended Data Fig. 5d–f). By histological analysis of Panc02 or KPC_1_ orthotopic tumors, we could consistently observe an increase in CD8^+^ T cell infiltration at the center of the tumor (Fig. 5f–h and Extended Data Fig. 5g).

Instead, in the KPC_3_ model, flow cytometry analysis revealed no differences in the number of CD8^+^ T cells and in the CD8^+^/T_reg_ cell ratio (Fig. 5a,b, right) but augmented CD69 and IFNγ expression in sgSlc4a4 tumors (Fig. 5i and Extended Data Fig. 5h). However, as in the other models, CD8^+^ T cells were more located at tumor center after sgSlc4a4 targeting and were substantially more abundant in the periphery as well (Fig. 5j and Extended Data Fig. 5i), suggesting that although the total number of CD8^+^ T cells did not change, these cells were better suited to enter into the tumor, and they were more activated.

**Extended Data Fig. 6:**
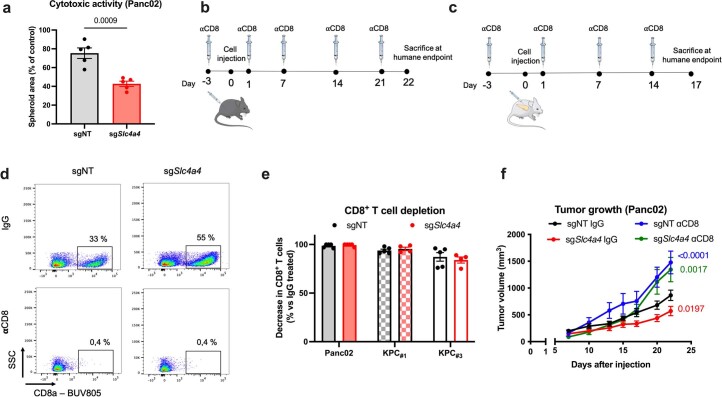
Effect of CD8^+^ T cell depletion following targeting of *Slc4a4* in cancer cells. **a**, Cytotoxic activity of CD8^+^ OT-1 T cells co-cultured with sgNT (n = 5) or sg*Slc4a4* (n = 5) Panc02 OVA spheroids. n represents independently harvested cells. **b**,**c**, Schematic overview of *in vivo* CD8^+^ T cell depletion in Panc02 (**b**) and KPC_#1/3_ (**c**) tumor models. Mice were treated with anti-CD8 (αCD8) depleting antibodies three days before tumor injection (−3). After subcutaneous Panc02 (**b**) or orthotopic KPC_#1/3_ (**c**) cell injection, αCD8 depleting antibodies were administered once per week (day 7, 14 and 21 after tumor injection). **d**, Representative flow cytometry plots of CD8^+^ T cell depletion within sgNT and sg*Slc4a4* tumors. **e**, Efficiency of depletion (FACS) of CD8^+^ T cells within sgNT (n = 5) and sg*Slc4a4* (n = 5) subcutaneous Panc02 (left), in sgNT (n = 5) and sg*Slc4a4* (n = 4) orthotopic KPC_#1_ (middle) and in sgNT (n = 5) and sg*Slc4a4* (n = 4) orthotopic KPC_#3_ tumors (right) upon αCD8 antibody treatment. Data are shown as percentage of depletion in αCD8-treated tumors, relative to the control IgG-treated groups. **f**, Growth of sgNT and sg*Slc4a4* Panc02 tumor subcutaneously injected in mice treated with an anti-CD8 depleting antibody (αCD8) or control IgG (IgG) (sgNT IgG n = 12, sg*Slc4a4* IgG n = 12, sgNT αCD8 n = 5, sg*Slc4a4* αCD8 n = 5). All statistics are referred to the sgNT-IgG condition. *P* value was assessed by unpaired, two-tailed Student’s t-test (**a**,**e**) and two-way ANOVA with Tukey’s multiple comparison test (**f**). Graphs show mean ± SEM. Source data

Improved activation of CD8^+^ T cells in sgSlc4a4 tumors was further corroborated by in vitro cytotoxic assays. When ovalbumin-expressing cancer cells were cocultured with OT-I T cells, we observed that OT-I T cells were able to kill more sgSlc4a4 cells than sgNT cancer cells (Fig. 5k, control), together with an increase in cytotoxicity markers such as IFNγ and granzyme B (GZMB; Extended Data Fig. 5j). This difference was abrogated by adding to the medium HCl or lactic acid but not by the supplementation of sodium lactate (Fig. 5k). The higher cytotoxic activity of CD8^+^ T cells toward sgSlc4a4 cancer cells was further confirmed in a coculture of T cells with tumor cell spheroids (Extended Data Fig. 6a). When CD8^+^ T cells were cultured in a conditioned medium derived from sgSlc4a4 cancer cells, they also displayed more robust in vitro proliferation (Fig. 5l). As above, differences in CD8^+^ T cell proliferation were lost after acidification of the conditioned medium from sgSlc4a4 cancer cells with HCl or lactic acid to match the parameters measured in the conditioned medium from sgNT cancer cells, while they were maintained after addition of sodium lactate (Fig. 5l). These results, in line with our metabolic data on pH and lactate metabolism, argue that sgSlc4a4 cancer cells alter the extracellular medium composition in a way to favor T cell proliferation and activation.

The major role of CD8^+^ T cell activation after *Slc4a4* targeting was further supported by the observation that CD8^+^ T cell depletion (Extended Data Fig. 6b–e) completely abolished the difference in tumor growth in the subcutaneous Panc02 and orthotopic KPC_1_ and KPC_3_ models (Fig. 5m–o and Extended Data Fig. 6f).

**Fig. 6:**
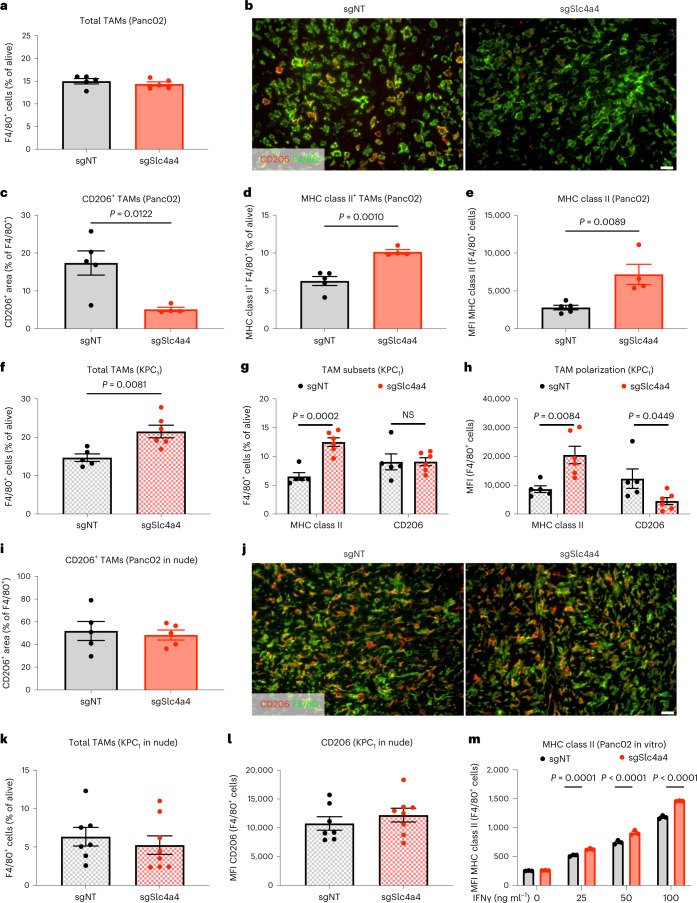
*Slc4a4* targeting affects TAMs only in presence of T cell-derived factors. **a**, Percentage of F4/80^+^ TAMs (FACS) in sgNT (*n* = 5) and sgSlc4a4 (*n* = 5) subcutaneous Panc02 tumors. **b**,**c**, Representative images (**b**) and quantification (**c**) of immunofluorescence stainings for CD206 (red) and F4/80 (green) in sgNT (*n* = 5) and sgSlc4a4 (*n* = 4) subcutaneous Panc02 tumors. **d**, Percentage of MHC class II^+^ TAMs (FACS) in sgNT (*n* = 5) and sgSlc4a4 (*n* = 4) subcutaneous Panc02 tumors. **e**, MFI of MHC class II in TAMs (FACS) in sgNT (*n* = 5) and sgSlc4a4 (*n* = 4) subcutaneous Panc02 tumors. **f**, Percentage of F4/80^+^ TAMs (FACS) in sgNT (*n* = 5) and sgSlc4a4 (*n* = 6) orthotopic KPC_1_ tumors. **g**, Percentage of MHC class II^+^ (left) and CD206^+^ (right) TAMs (FACS) in sgNT (*n* = 5) and sgSlc4a4 (*n* = 6) orthotopic KPC_1_ tumors. **h**, MFI of MHC class II (left) and CD206 (right) in TAMs (FACS) in sgNT (*n* = 5) and sgSlc4a4 (*n* = 6) orthotopic KPC_1_ tumors. **i**,**j**, Quantification (**i**) and representative images (**j**) of immunofluorescence stainings for CD206 (red) and F4/80 (green) in sgNT (*n* = 5) and sgSlc4a4 (*n* = 5) subcutaneous Panc02 tumors injected in nude mice. **k**, Percentage of F4/80^+^ TAMs (FACS) in sgNT (*n* = 7) and sgSlc4a4 (*n* = 8) orthotopic KPC_1_ tumors injected in nude mice. **l**, MFI of CD206 in TAMs (FACS) in sgNT (*n* = 7) and sgSlc4a4 (*n* = 8) orthotopic KPC_1_ tumors injected in nude mice. **m**, MFI of MHC class II in BMDMs (FACS) cocultured with sgNT (*n* = 3) and sgSlc4a4 (*n* = 3) Panc02 cells in the absence or presence of different concentrations of IFNγ (25, 50 and 100 ng ml^–1^). *n* represents independently collected cells (**m**). *P* value was assessed by unpaired, two-tailed Student’s *t*-test (**a**, **c**–**i**, **k** and **l**) and two-way ANOVA with Sidak’s multiple comparison test (**m**). Graphs show mean ± s.e.m.; scale bars, 20 μm (**b** and **j**). Source data

**Extended Data Fig. 7:**
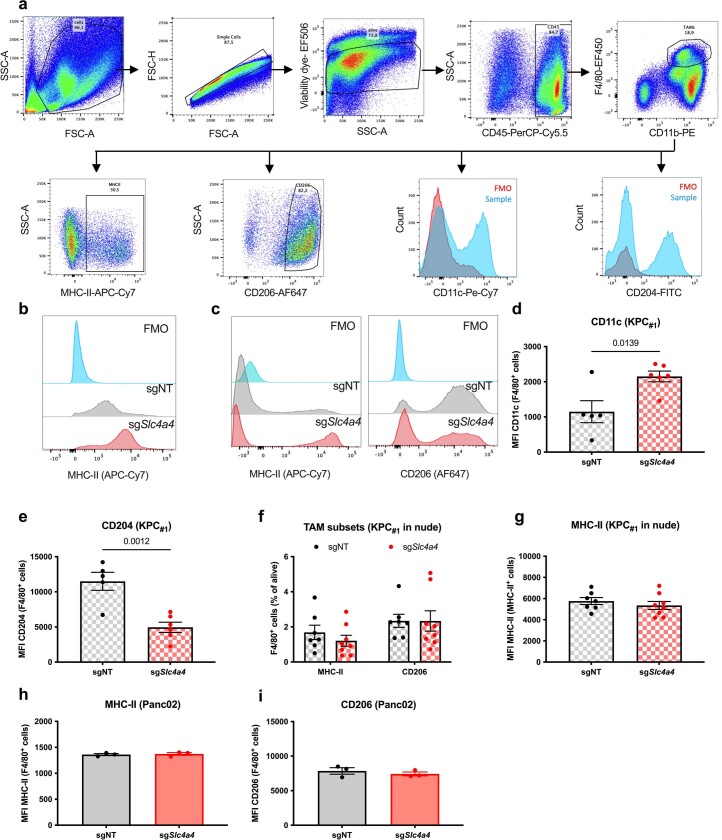
*Slc4a4* targeting does not affect *per se* macrophage polarization *in vitro*. **a**, Gating strategy for TAM compartment (numbers represent percentage of cells out of parental population). **b**, Representative histograms of the MFI of MHCII in F4/80+ cells (FACS) in sgNT and sgSlc4a4 subcutaneous Panc02 tumors. **c**, Representative histograms of the MFI of MHCII (left) and CD206 (right) in F4/80+ cells (FACS) in sgNT and sgSlc4a4 orthotopic KPC#1 tumors. **d**,**e**, MFI of CD11c (**d**) and CD204 (**e**) in TAMs (FACS) in sgNT (n = 5) and sgSlc4a4 (n = 6) orthotopic KPC#1 tumors. **f**, Percentage of MHC-II + (left) and CD206 + (right) TAMs (FACS) in sgNT (n = 7) and sgSlc4a4 (n = 8) orthotopic KPC#1 tumors injected in nude mice. **g**, MFI of MHC-II in TAMs (FACS) in sgNT (n = 7) and sgSlc4a4 (n = 8) orthotopic KPC#1 tumors injected in nude mice. **h**,**i**, MFI of MHC-II (**h**) and CD206 (**i**) in BMDMs (FACS) co-cultured with sgNT (n = 3) and sgSlc4a4 (n = 3) Panc02 cells. n represents independently harvested cells (**h**,**i**). P value was assessed by unpaired, two-tailed Student’s t-test (**d**-**i**). Graphs show mean ± SEM. Source data

Tumor-associated macrophages (TAMs) also respond to changes in environmental acidity^4,37^. While the total number of TAMs was not affected (Fig. 6a and Extended Data Fig. 7a), subcutaneous sgSlc4a4 Panc02 tumors displayed less protumoral/M2-like macrophages^38^, defined as positive for both F4/80 and CD206 (Fig. 6b,c), and more antitumoral/M1-like macrophages, defined as positive for F4/80 and major histocompatibility class II (MHC class II; Fig. 6d), also confirmed by enhanced mean fluorescence intensity (MFI) of MHC class II in F4/80^+^ cells from sgSlc4a4 versus sgNT tumors (Fig. 6e and Extended Data Fig. 7b). In KPC_1_ orthotopic tumors, sgSlc4a4 targeting led to an increased number of total macrophages (Fig. 6f) due to more M1-like MHC class II^+^ macrophages (Fig. 6g, left), expressing higher levels of MHC class II and CD11c (Fig. 6h, left, and Extended Data Fig. 7c,d), while M2-like CD206^+^ macrophages were similar in numbers but with reduced levels of CD206 and CD204 (Fig. 6g,h, right, and Extended Data Fig. 7e). In subcutaneous Panc02 or in orthotopic KPC_1_ tumors grafted in nude mice, both the total number of TAMs and their polarization were similar in sgNT and sgSlc4a4 tumors (Fig. 6i–l and Extended Data Fig. 7f,g), suggesting that that the differences observed in TAM polarization in immunocompetent mice requires the involvement of T cells rather than resulting from a direct effect of the pH modulation induced by sgSlc4a4 cancer cells. Consistently, in vitro analysis of bone marrow-derived macrophages (BMDMs) cocultured with sgNT and sgSlc4a4 Panc02 cells did not show any differences in the polarization markers (Extended Data Fig. 7h,i), but the presence of sgSlc4a4 cancer cells, along with different concentrations of IFNγ (predominantly secreted by T cells^39,40^) sensitized BMDMs to an M1-like phenotypic switch (Fig. 6m). Overall, this suggests that an improvement of the pH_e_ is per se not sufficient to skew TAMs toward an M1-like phenotype but ultimately requires the presence of T cell-derived signals, that is, IFNγ.

These data support the idea that decreased growth of sgSlc4a4 tumors in immunocompetent mice is due to improved activation and reduced suppression of the immune system rather than a growth defect of cancer cells.

### *Ldha* overexpression counteracts *Slc4a4* targeting

**Fig. 7:**
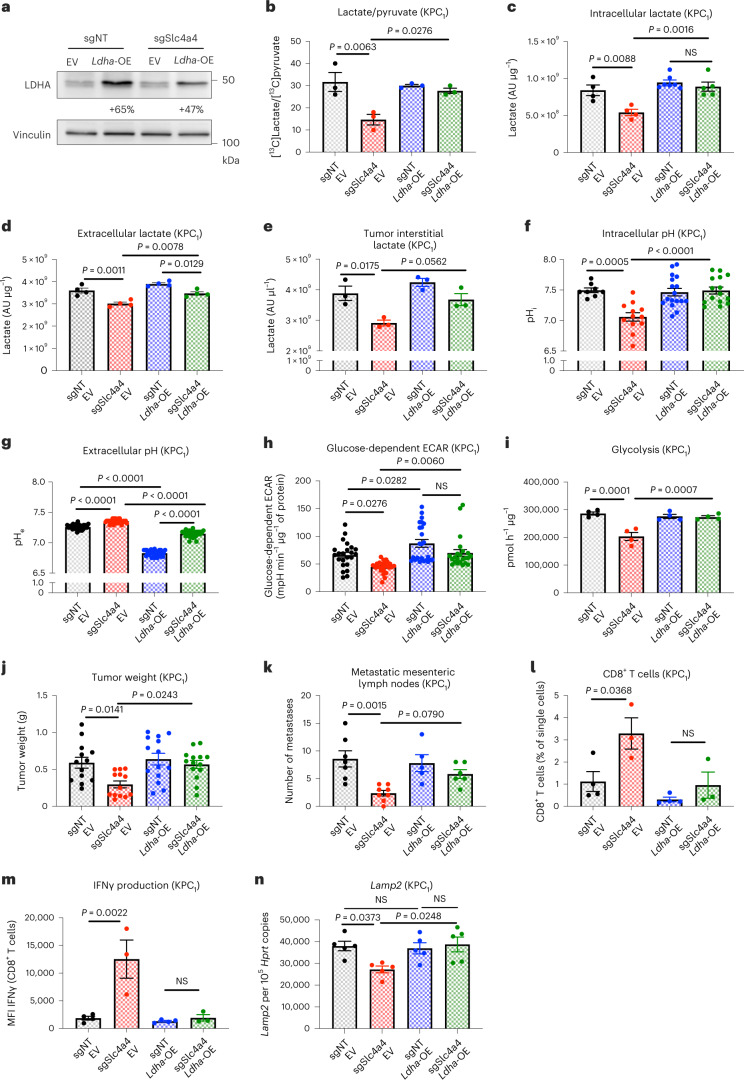
Overexpression of *Ldha* counteracts the effects of *Slc4a4* targeting. **a**, LDHA protein levels assessed by western blotting analysis in sgNT and sgSlc4a4 KPC_1_ cells overexpressing an empty vector (EV) or *Ldha* (*Ldha*-OE). **b**, [^13^C]Lactate:[^13^C]pyruvate ratio in sgNT empty vector (*n* = 3), sgSlc4a4 empty vector (*n* = 3), sgNT *Ldha*-OE (*n* = 3) and sgSlc4a4 *Ldha*-OE (*n* = 3) KPC_1_ cells. **c**,**d**, Intracellular (**c**) and extracellular (**d**) lactate concentration measured by LC–MS in sgNT empty vector (*n* = 4 and 4), sgSlc4a4 empty vector (*n* = 4 and 4), sgNT *Ldha*-OE (*n* = 7 and 4) and sgSlc4a4 *Ldha*-OE (*n* = 5 and 4) KPC_1_ cells. **e**, Lactate concentration measured by LC–MS in tumor interstitial fluid of sgNT empty vector (*n* = 3), sgSlc4a4 empty vector (*n* = 3), sgNT *Ldha*-OE (*n* = 3) and sgSlc4a4 *Ldha*-OE (*n* = 3) KPC_1_ tumors. **f**,**g**, Intracellular (**f**) and extracellular (**g**) pH of sgNT empty vector (*n* = 8 and 30), sgSlc4a4 empty vector (*n* = 12 and 30), sgNT *Ldha*-OE (*n* = 17 and 30) and sgSlc4a4 *Ldha*-OE (*n* = 15 and 30) KPC_1_ cells. **h**, Glucose-dependent ECAR of sgNT empty vector (*n* = 23), sgSlc4a4 empty vector (*n* = 24), sgNT *Ldha*-OE (*n* = 24) and sgSlc4a4 *Ldha*-OE (*n* = 23) KPC_1_ cells. **i**, ^3^H_2_O release from [^3^H]glucose in sgNT empty vector (*n* = 4), sgSlc4a4 empty vector (*n* = 4), sgNT *Ldha*-OE (*n* = 4) and sgSlc4a4 *Ldha*-OE KPC_1_ (*n* = 4) cells. **j**, Weight of sgNT empty vector (*n* = 13), sgSlc4a4 empty vector (*n* = 13), sgNT *Ldha*-OE (*n* = 14) and sgSlc4a4 *Ldha*-OE (*n* = 14) orthotopic KPC_1_ tumors. Data show a pool of two independent experiments. **k**, Macroscopic metastatic mesenteric lymph nodes of sgNT empty vector (*n* = 7), sgSlc4a4 empty vector (*n* = 8), sgNT *Ldha*-OE (*n* = 5) and sgSlc4a4 *Ldha*-OE (*n* = 6) orthotopic KPC_1_ tumors. **l**, Percentage of CD8^+^ T cells (FACS) in sgNT empty vector (*n* = 4), sgSlc4a4 empty vector (*n* = 3), sgNT *Ldha*-OE (*n* = 4) and sgSlc4a4 *Ldha*-OE (*n* = 3) orthotopic KPC_1_ tumors. **m**, MFI of IFNγ in CD8^+^ T cells (FACS) in sgNT empty vector (*n* = 4), sgSlc4a4 empty vector (*n* = 3), sgNT *Ldha*-OE (*n* = 4) and sgSlc4a4 *Ldha*-OE (*n* = 3) orthotopic KPC_1_ tumors. **n**, *Lamp2* expression assessed by RT–qPCR analysis in sgNT empty vector (*n* = 5), sgSlc4a4 empty vector (*n* = 5), sgNT *Ldha*-OE (*n* = 5) and sgSlc4a4 *Ldha*-OE (*n* = 5) orthotopic KPC_1_ tumors. Data were normalized by protein content (**c**, **d**, **h** and **i**). The experiment in **a** was repeated three times with similar results. *n* represents independently collected cells (**b**–**i**). *P* value was assessed by two-way ANOVA with Tukey’s multiple comparison test (**b**–**n**). Graphs show mean ± s.e.m. Source data

Previous findings show that pH regulation of LDHA activity is overcome by increased enzyme concentration^41^, which consequently reinforces the entire glycolytic flux^42,43^. To assess whether the effect of sgSlc4a4 on PDAC progression was mediated by an impairment in lactate production that, in turn, would further ameliorate the pH_e_ and thus immune functions^3,44^, we overexpressed *Ldha* (*Ldha*-OE) or used an empty vector as control in KPC_1_ cells (Fig. 7a). By using [^13^C]pyruvate, we proved that lactate generation was increased in sgSlc4a4 cells following the overexpression of *Ldha* (Fig. 7b). Consistently, intracellular and extracellular lactate concentration in *Ldha-*OE sgSlc4a4 cells was raised up to the same level as in sgNT cells, whereas *Ldha* overexpression in sgNT cells minimally affected lactate levels, arguing that lactate production is almost at saturation in control cells (Fig. 7c,d). A similar pattern was observed when measuring the interstitial lactate concentration in tumors (Fig. 7e). In addition, *Ldha* overexpression in sgSlc4a4 cells led to a pH_i_ increase to the same value measured in sgNT cells and a simultaneous pH_e_ decrease (Fig. 7f,g). Similar results were observed when measuring two independent readouts of glycolysis, that is, glucose-dependent ECAR and tritiated water (^3^H_2_O) release out of [^3^H]glucose (Fig. 7h,i). These data suggest that *Ldha* overexpression is able to increase lactate production under conditions of SLC4A4 inhibition, restoring lactate and pH levels back to the control condition. Phenotypically, *Ldha* overexpression abolished the difference in tumor and metastatic growth between the sgNT and sgSlc4a4 conditions (Fig. 7j,k). The number of CD8^+^ T cells and their IFNγ production were both reduced to control levels in *Ldha*-OE sgSlc4a4 tumors (Fig. 7l,m). Consistent with the in vitro data, increased *Lamp2* expression indirectly indicated a rescued acidosis in *Ldha*-OE sgSlc4a4 tumors (Fig. 7n). Thus, the rewiring of the immune system induced by *Slc4a4* targeting is due to the mitigation of the acidosis in the TME.

### *Slc4a4* targeting improves ICB efficacy

**Fig. 8:**
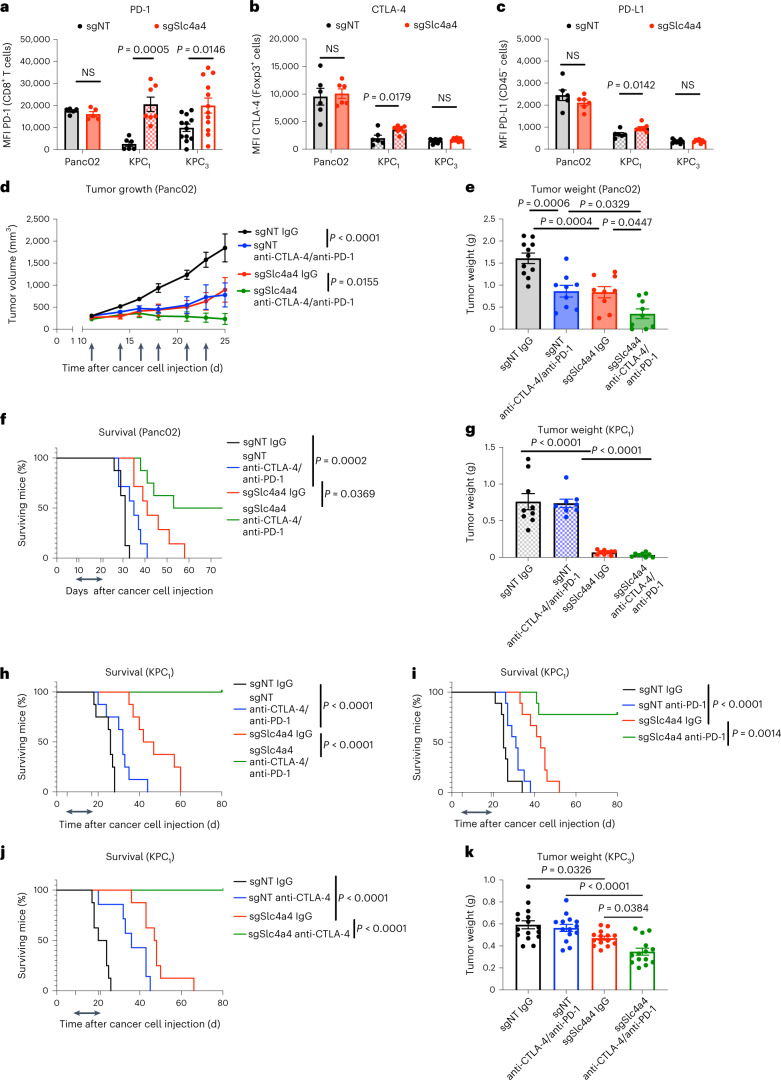
*Slc4a4* targeting overcomes immunotherapy resistance. **a**, MFI of PD-1 in CD8^+^ T cells in sgNT (*n* = 5, 6 and 12) and sgSlc4a4 (*n* = 5, 7 and 12) subcutaneous Panc02 (left) and orthotopic KPC_1_ (middle) and KPC_3_ tumors (right). **b**, MFI of CTLA-4 in Foxp3^+^ T cells in sgNT (*n* = 6, 6 and 8) and sgSlc4a4 (*n* = 6, 7 and 8) subcutaneous Panc02 (left) and orthotopic KPC_1_ (middle) and KPC_3_ tumors (right). **c**, MFI of PD-L1 in CD45^–^ cells in sgNT (*n* = 6, 6 and 8) and sgSlc4a4 (*n* = 6, 7 and 8) subcutaneous Panc02 (left) and orthotopic KPC_1_ (middle) and KPC_3_ tumors (right). **d**, Growth of sgNT and sgSlc4a4 subcutaneous Panc02 tumors treated with anti-PD-1 and anti-CTLA-4 (sgNT-IgG *n* = 6, sgNT-anti-PD-1/anti-CTLA-4 *n* = 6, sgSlc4a4-IgG *n* = 6, sgSlc4a4-anti-PD-1/anti-CTLA-4 *n* = 5). **e**, Weight of sgNT and sgSlc4a4 subcutaneous Panc02 tumors treated with anti-PD-1 and anti-CTLA-4 (sgNT-IgG *n* = 11, sgNT-anti-PD-1/anti-CTLA-4 *n* = 9, sgSlc4a4-IgG *n* = 9, sgSlc4a4-anti-PD-1/anti-CTLA-4 *n* = 9). **f**, Survival curve of sgNT and sgSlc4a4 subcutaneous Panc02 tumor-bearing mice treated with anti-PD-1 and anti-CTLA-4 (sgNT-IgG *n* = 8, sgNT-anti-PD-1/anti-CTLA-4 *n* = 7, sgSlc4a4-IgG *n* = 7, sgSlc4a4-anti-PD-1/anti-CTLA-4 *n* = 8). **g**, Weight of sgNT and sgSlc4a4 orthotopic KPC_1_ tumors treated with anti-PD-1 and anti-CTLA-4 (sgNT-IgG *n* = 9, sgNT-anti-PD-1/anti-CTLA-4 *n* = 8, sgSlc4a4-IgG *n* = 9, sgSlc4a4-anti-PD-1/anti-CTLA-4 *n* = 8). **h**, Survival curve of sgNT and sgSlc4a4 orthotopic KPC_1_ tumor-bearing mice treated with anti-PD-1 and anti-CTLA-4 (sgNT-IgG *n* = 8, sgNT-anti-PD-1/anti-CTLA-4 *n* = 8, sgSlc4a4-IgG *n* = 8, sgSlc4a4-anti-PD-1/anti-CTLA-4 *n* = 8). **i**, Survival curve of sgNT and sgSlc4a4 orthotopic KPC_1_ tumor-bearing mice treated with anti-PD-1 (sgNT-IgG *n* = 9, sgNT-anti-PD-1 *n* = 9, sgSlc4a4-IgG *n* = 9, sgSlc4a4-anti-PD-1 *n* = 9). **j**, Survival curve of sgNT and sgSlc4a4 orthotopic KPC_1_ tumor-bearing mice treated with anti-CTLA-4 (sgNT-IgG *n* = 8, sgNT-anti-CTLA-4 *n* = 8, sgSlc4a4-IgG *n* = 8, sgSlc4a4-anti-CTLA-4 *n* = 8). **k**, Weight of sgNT and sgSlc4a4 orthotopic KPC_3_ tumors treated with anti-PD-1 and anti-CTLA-4 (sgNT-IgG *n* = 16, sgNT-anti-PD-1/anti-CTLA-4 *n* = 14, sgSlc4a4-IgG *n* = 14, sgSlc4a4-anti-PD-1/anti-CTLA-4 *n* = 14). Data in **e** and **k** are representative of a pool of two independent experiments. Treatment regimen is indicated by the arrows (mice were treated three times per week with up to six injections; **d**, **f** and **h**–**j**). *P* value was assessed by unpaired, two-tailed Student’s *t*-test (**a**–**c**), two-way ANOVA with Sidak’s multiple comparison test (**d**), two-way ANOVA with Tukey’s multiple comparison test (**e**, **g** and **k**) and log-rank (Mantel–Cox) test (**f** and **h**–**j**). Graphs show mean ± s.e.m. Source data

**Extended Data Fig. 8:**
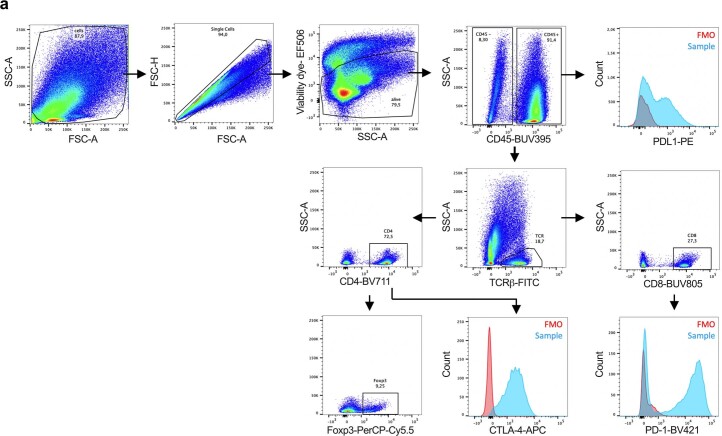
Immune check-point molecules. **a**, Gating strategy for immune checkpoint molecules (numbers represent percentage of cells out of parental population).

Despite the reinvigorated antitumoral immune response after *Slc4a4* targeting, in the Panc02 model, expression of programmed cell death protein 1 (PD-1) in CD8^+^ T cells and cytotoxic T lymphocyte-associated protein 4 (CTLA-4) in T_reg_ cells (considered the most representative cellular compartments for the expression of these immune checkpoints^45^) and programmed death ligand 1 (PD-L1) levels in cancer cells in vivo were still high and comparable in sgNT versus sgSlc4a4 Panc02 tumors (Fig. 8a–c, left, and Extended Data Fig. 8a). The same analysis in KPC_1_ tumors, instead, revealed an increased expression of these three markers probably due to the stronger CD8^+^ T cell activation and overall antitumoral phenotype (Fig. 8a–c, middle). In the KPC_3_ model where the antitumoral effect was milder, we could observe an increase only in PD-1 expression by CD8^+^ T cells (Fig. 8a–c, right).

Based on these results, we speculated that the combination of *Slc4a4* targeting and immunotherapy could further tackle tumor progression. For this purpose, sgSlc4a4 and sgNT tumors (Panc02 or KPC_1_ and KPC_3_) were treated with the ICB anti-PD-1 and anti-CTLA-4 (ref. ^46^). Treatment was given in six injections spread over 2 weeks (starting on day 5 for KPC tumors and from 200-mm^3^ tumor volume for Panc02 tumors). In the subcutaneous setting with Panc02 tumors, anti-PD-1/anti-CTLA-4 treatment resulted in a synergic effect, with *Slc4a4* deletion leading to tumor regression or to a static disease (Fig. 8d,e), overall offering an increased survival (Fig. 8f).

In the orthotopic KPC_1_ model where *Slc4a4* targeting per se already displayed a strong reduction in tumor growth, we could observe a trend in tumor growth reduction by adding the treatment with anti-PD-1 and anti-CTLA-4 together (Fig. 8g). When looking at survival, deletion of *Slc4a4* alone increased life expectancy compared to that observed in the sgNT group. The sgNT group had a median survival of 32 d, displaying an increased median survival of only 6 d compared to the corresponding non-treated group (Fig. 8h). Strikingly, sgSlc4a4 tumor-bearing mice treated with ICB were all alive and overtly healthy at day 80. Necropsy did not reveal any sign of tumor nor the presence of metastatic mesenteric lymph nodes, indicating a complete tumor regression and a synergic effect between *Slc4a4* deletion and ICB treatment. Given the strong effect of ICB in this model, we investigated the effect of the single treatment anti-PD-1 or anti-CTLA-4. Also in this case, we obtained a striking survival advantage in sgSlc4a4 tumor-bearing mice treated with a single ICB (Fig. 8i,j). Finally, while sgNT KPC_3_ tumors were completely resistant to anti-PD-1/anti-CTLA-4 treatment, *Slc4a4* targeting sensitized these tumors to ICB, without, however, leading to a whole regression (Fig. 8k).

Overall, these data show that inhibition of SLC4A4 in combination with ICB strongly reduces tumor growth and cancer aggressiveness, paving the way toward a possible therapeutic strategy to overcome PDAC resistance to immunotherapy.

## Discussion

In solid tumors, extracellular acidity is one of the main features of the TME^47^, which impedes an effective antitumoral response^1^ and favors metastatic dissemination^2,48^. In normal cell physiology, bicarbonate transporters are involved in maintaining the optimal acid/base equilibrium for the cell^15^. In the current study, we have hypothesized that bicarbonate transport ability gets hijacked by cancer cells, and this contributes to increase the acidification of the TME and, at the same time, to maintain an optimal pH_i_ in a harsh environment. To better elucidate the underlying mechanisms of pH regulation by this family of transporters during tumor progression, we focused on SLC4A4, which, according to both published and in-house single-cell RNA-seq data from individuals with PDAC, is the most expressed bicarbonate transporter in this tumor and is predominantly expressed by the ductal epithelial compartment.

**Extended Data Fig. 9:**
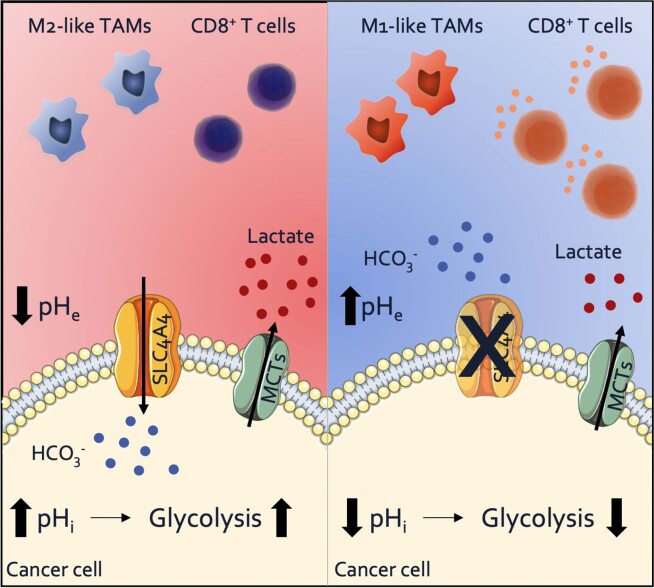
Scheme illustrating the role of SLC4A4 in PDAC progression. SLC4A4, the most abundant bicarbonate transporter in PDAC and almost exclusively expressed in the cancer cell compartment, favors the accumulation of intracellular bicarbonate. Its activity maintains an optimal intracellular pH (pH_i_), thus preventing acidosis-dependent glycolysis inhibition, and, in a feed forward loop, further decreasing the extracellular pH (pH_e_) because of the excretion of lactate in the extracellular space. Thus, SLC4A4 proficient tumors are characterized by an acidic TME and high levels of lactate that dampen CD8^+^ T cell activation and favor M2-like skewing of TAMs. On the other hand, genetic or pharmacological inhibition of SLC4A4 decreases bicarbonate uptake by the cancer cells, thus preventing TME acidosis while reducing the pH_i_, which inhibits glycolysis in a pH-dependent manner, and consequently impairs lactate production and export. Overall, SLC4A4 targeting decreases acidosis and extracellular lactate boosting anti-tumoral CD8^+^ T cell response and M1-like skewing, ultimately harnessing tumor progression and overcoming resistance to ICB.

In vitro and in vivo analyses showed that SLC4A4 contributes to the modulation of pH_e_ during PDAC progression. The inhibition of this transporter impedes the uptake of bicarbonate by cancer cells, leading to its accumulation in the extracellular space and, thereby, increasing the pH_e_. Despite that pH is often assumed but not always measured in vivo, in this study, we were able to confirm our in vitro findings and show that *Slc4a4* targeting in cancer cells importantly affects the TME acidity using MRS technology. The intracellular buffer ability of SLC4A4-deficient cells was also reduced, resulting in a lower pH_i_, which we link to a decrease in glycolysis that, in turn, contributes to further ameliorate the pH of the extracellular milieu through reduced lactate production and, consequently, its reduced export coupled with protons. On the contrary, it is likely that SLC4A4-proficient cancer cells are able to preserve an optimal pH_i_ by sustaining bicarbonate import and thus preventing acidosis-dependent glycolysis inhibition^28,30–32^. In this way, cancer cells install a loop in which bicarbonate sequestered from the extracellular space not only reduces the pH_e_ but also favors glycolysis and consequently lactate accumulation and secretion, which, in turn, further decreases the pH_e_. Through this mechanism, PDAC cells maintain and fuel an acidic TME (Extended Data Fig. 9). Of note, the overexpression of LDHA is able to rescue the observed phenotype, even if the precise mechanism by which increased expression of LDHA overcomes pH-dependent inhibition of its activity needs to be further studied. Notably, another sodium bicarbonate cotransporter controlling acid extrusion, SLC4A7, favors tumor development and progression in breast cancer, highlighting the relevance of bicarbonate import during cancer progression^49^.

Because we ruled out any difference in the proliferative rate, cell cycle and apoptosis of pancreatic cancer cells in vitro or an effect on tumor growth in immunocompromised mice, these data indicate that *Slc4a4* targeting in cancer cells imposes a cell-extrinsic alteration of the TME that hinders tumor growth and metastasis through the immune system. This might appear in contradiction with in vitro evidence performed in breast, prostate and colorectal cancer cells where *Slc4a4* silencing negatively affects cancer cell proliferation^16–18^. Unlike other tissues, normal pancreatic duct epithelial cells are exposed to a heterogenous and fluctuating gradient of pH (for example, alkaline in the apical side while acidic in the basolateral side), and, therefore, they might better adapt to pH modifications^50^.

Tumor progression is strongly affected by the cellular composition of the TME. In particular, immune cells can display an antitumoral phenotype, although usually restrained, or protumoral features, which are sustained by cancer cells and environmental factors^51^. The most powerful antitumoral immune cells are CD8^+^ cytotoxic T cells that are able to recognize and kill cancer cells, although they are generally scarce and anergic/exhausted in the TME. Several studies have shown that low pH_e_ and lactate accumulation are both responsible for impaired T cell activation and GZMB and IFNγ production in human and mouse systems^5,7,8,52^. These effects can be attributed to lactate-mediated impairment of JAK3/STAT5 and MAPK phosphorylation required for a proper response to T cell antigen receptor stimulation^7,52^. Moreover, because activated T cells rely predominantly on glycolysis, high extracellular levels of lactate impair lactate export, leading to a metabolic blockade^8,28,30–32^. Consistently, we found that the acidification of the TME sustained by SLC4A4 activity blunts the effectiveness of a proper antitumoral CD8^+^ T cell response. Our data show also that SLC4A4 targeting installs a proinflammatory and less immunosuppressive macrophage phenotype only in the concomitant presence of T cells. The evidence that a decrease in lactic acid or, in general, an increase of the pH in the TME can per se revert the M2-like TAM phenotype into a more M1-like phenotype is sparse and relies on experimental proofs obtained over a wide range of pH/lactate changes^4,53^. Our in vitro and in vivo data strongly support the idea that a mild deacidification of the TME sensitizes TAMs to leukocyte-derived signals, such as IFNγ, reducing the threshold of an M1-like phenotypic switch in response to this cytokine.

PDAC is one of the most aggressive and lethal cancer types, with a 5-year survival rate lower than 10% (ref. ^54^). Standard chemotherapy has failed to provide individuals with a promising treatment option, and, although immunotherapy was proven to be efficient in different cancer types such as melanoma or renal cell carcinoma, PDAC remains completely resistant^55^. In line with previous findings showing that neutralizing the acidification of the TME and/or increasing IFNγ production by CD8^+^ T cells enhances the response to immunotherapy in different tumors, such as melanoma and breast cancer^5,10,56^, we have demonstrated that inhibition of SLC4A4 can sensitize PDAC to ICB treatment, leading to complete or partial regression of orthotopic KPC tumors and longer survival, further underlining the therapeutic potential of SLC4A4 blockade.

In light of the importance of pH regulation for tumor progression, preventing tumor acidity has been already evaluated as a therapeutic option. So far, the strategies to ameliorate the tumor pH have been mainly directed to decrease extracellular lactate concentration via the inhibition of glycolysis and therefore lactate production (that is, LDHA inhibitors) or via the inhibition of lactate secretion (that is, MCT1 inhibitors)^3,10,57^. These approaches, however, do not affect the metabolism of cancer cells only but impact the metabolic machinery of antitumoral immune cells present in the TME. It is now well known that the proliferation and activation of antitumoral immune cells, like M1-like macrophages and especially CD8^+^ T cells, are supported by a strong glycolytic metabolism^58^. Although the previous strategies mitigate the acidity of the TME, they also display a detrimental effect on the immune response of these cells^8,59^. Moreover, efficient pharmacological LDHA inhibition has been proven to be challenging, as assessed in several cell-based assays, or, ultimately, LDHA-targeting molecules have unveiled important pharmacokinetic issues when tested in vivo^60^. Aiming to a rewiring of the TME, at least in PDAC, SLC4A4-targeted strategies offer the advantage to tackle bicarbonate transport in cancer cells only and to open a therapeutic window in which ICB can exert their effects. In this sense, the restricted use of SLC4A4 inhibitors within this (short) temporal window could minimize possible side effects linked to the block of SLC4A4 in other organs, such as the kidney and brain^15^. Although at the moment this remains an appealing hypothesis, extensive drug development programs are warranted in the future.

In conclusion, SLC4A4 targeting leads simultaneously to the accumulation of bicarbonate and to the reduction of lactate in the tumor milieu. Overall, these metabolic changes are able to restore the antitumor effector functions of tumor-infiltrating CD8^+^ T cells and TAMs. These results could pave the way toward SLC4A4-based therapeutic strategies that mitigate tumor acidosis, abate immunosuppression, increase CD8^+^ T cell fitness and sensitize PDAC to the current immunotherapeutic regimens, an unmet clinical need for far.

## Methods

### Animals

All experimental animal procedures were approved by the Institutional Animal Care and Research Advisory Committee of the KU Leuven (P226/2017). Mice were maintained under pathogen-free and temperature- and humidity-controlled conditions with a 12-h light/12-h dark cycle and received normal chow (ssniff, R/M-H). Animals were removed from the study and killed if any signs of pain and distress were detected or if the tumor volume reached 2,000 mm^3^. The maximal tumor size was not exceeded in all reported studies.

FVB, C57BL6/N and NMRI nu/nu nude mice were purchased from Envigo. Rag2/OT-1 mice were purchased from Taconic. All mice used for tumor experiments were females between 8 and 12 weeks old.

### Human PDAC samples

For RNA-seq, human PDAC samples were obtained from 10 treatment-naive individuals (7 females and 3 males) with a median age of 66.5 (range of 47–81 years) after signed informed consent. Resection material was collected from primary tumors during surgery. The presence of adenocarcinoma was proven by histopathology. Samples were processed and studied using single-cell RNA-seq with 10x Genomics. The median tumor diameter of PDAC primary tumors was 30 mm (range of 14–61). According to the tumor, node, metastasis (TNM) classification (UICC 8th edition), three individuals were stage I, two were stage II, one was stage III and four were stage IV. IHC samples were obtained from seven individuals (two females and five males) with a median age 64 (range 42–73 years). The study was approved by the Ethical Committee of the University Hospitals KU Leuven (ML3452).

### Cell lines

The mouse PDAC Panc02 cell line was kindly provided by B. Wiedenmann (Charité, Berlin) and cultured in DMEM (Gibco) supplemented with 10% of fetal bovine serum (FBS; Gibco) and 1% penicillin/streptomycin (pen/strep; Gibco). The mouse KPC cell lines were kindly provided by the Hanahan laboratory at the École Polytechnique Fédérale de Lausanne. KPC cells were generated from FVB mice carrying different genetic mutations P48Cre/*Kras*^G12D^/*Trp53*^LSL R172H^. KPC_1_ cells were generated from male FVB/n mice, whereas KPC_2_ and KPC_3_ cells were from female FVB/n mice. The cells were cultured in RPMI medium (Gibco; 10% FBS and 1% pen/strep). All the cells were grown at 37 °C in a humidified 5% CO_2_ incubator.

### In vivo experiments

C57BL6/N mice were injected subcutaneously in the flank with 4 × 10^6^ Panc02 cells in 200 μl. Tumor growth was monitored by measuring the perpendicular diameters of tumors every other day. C57BL6/N and FVB mice were injected orthotopically in the pancreas with 1 × 10^6^ Panc02 cells and 1 × 10^4^ KPC cells, respectively, in 20 μl. Body weight was monitored. Mice were killed at a humane endpoint. Metastatic mesenteric lymph nodes were quantified as previously reported^61,62^. For hydrodynamic injection, FVB mice were injected via the tail vein with 5 × 10^5^ KPC cells in a volume corresponding to 10% of the body weight. Body weight was monitored, and mice were killed at a humane endpoint. Immunotherapy treatment was done intraperitoneally (i.p.) with 10 mg per kg (body weight) of control IgG, anti-PD-1 or anti-CTLA-4 (three times per week). For CD8^+^ T cell depletion, mice were injected i.p. with anti-CD8 (10 mg per kg) 3 d before tumor inoculation and then one time per week. For in vivo SLC4A4 inhibition, mice were treated with 15 mg per kg of DIDS (Sigma) i.p. twice daily for 10 d.

The antibodies used included rat serum IgG (Sigma-Aldrich, I4131), Ultra-LEAF purified anti-mouse PD-1 (CD279; BioLegend, 96167, RMP1–14), InVivoMAb anti-mouse CTLA-4 (CD152; BioCell, BE0164, 9D9) and InVivoMAb anti-mouse CD8α (BioCell, BE0004-1, 53-6.7).

### RNA-seq data processing

Single-cell datasets generated in-house were processed using the CellRanger 3.1.0 pipeline and mapped to the human reference genome (GrCh38). The output raw feature–barcode matrix was imported into ScanPy (v1.6.0). Cells with low quality (<200 genes per cell) and rare genes (expressed by less than 30 cells) were removed. Dying cells with a mitochondrial percentage greater than 20% were also excluded; 19,309 cells were analyzed. The following are the number of cells per individual: PAN005_PAN (1,269), PAN006 (459), PAN008 (2,062), PAN009 (1,297), PAN011 (3,901), PAN015_PAN (1,632), PAN016 (722), PAN017 (2,351), PAN018_PAN (2,207) and PAN021_T (3,409). Cell counts were normalized using scanpy.pp.normalize_per_cell (scaling factor of 10,000), and gene expression was scaled to unit variance and a mean value of 0 using scanpy.pp.scale. Dimensionality reduction of the data was done by principal-component analysis using the scanpy.tl.pca function. The scanpy.pl.pca_variance_ratio plot was used to determine the inflection point after which no remarkable change in the variance was observed. The neighborhood graph for clustering was calculated using scanpy.pp.neighbors, while the scanpy.tl.leiden function was used to cluster the cells using Leiden clustering. Differentially expressed genes across the Leiden clusters were determined using scanpy.tl.rank_genes_groups and were used to check cluster validity.

Normalized bulk RNA-seq expression data from the The Cancer Genome Atlas (TCGA) Pancreatic Cancer (PAAD) dataset (*n* = 182) were downloaded from the UCSC Xena Resource (http://xena.ucsc.edu/). The R package ggplot2 was used to compare *SLC4A4* expression in primary tumors versus adjacent tissue.

### Histology and immunostainings

Human PDAC samples were cut at 5 µm using the BOND Max system (Leica Microsystems) and a BOND Polymer Refine Detection kit (DS9800). For SLC4A4 immunohistochemistry (IHC), samples were dewaxed with the BOND dewax solution (AR9222), incubated for 20 min in BOND epitope retrieval solution 2 (AR9640; pH 9) and blocked for 5 min for endogenous peroxidase. Slides were incubated with primary antibody to SLC4A4/NBC (1:5,000; Abcam, ab187511), followed by a horseradish peroxidase (HRP)-labeled secondary antibody for 30 min each. 3,3′-Diaminobenzidine chromogen (DAB) was added for visualization (10 min). Slides were rehydrated, counterstained with hematoxylin and mounted. The expression pattern of SLC4A4 was determined by a specialized pathologist (T.R.). Images were captured using the Leica DFC290-HD Digital FireWire camera (Leica Microsystems).

For immunofluorescence stainings and IHC of cytokeratin-19 (CK19), tumor and liver samples were stained as previously described^63^. The following primary antibodies were used: rat anti-F4/80 (1:100; Serotec, MCA497F), rat anti-CD34 (1:100; BD Pharmigen, 553731, RAM34), mouse anti-MMR/CD206 (1:100; R&D Systems, AF2535), rabbit anti-fluorescein isothiocyanate (FITC; 1:200; Serotec, 4510–7604), rabbit anti-CK19 (1:100; Abcam, ab15463), rabbit anti-CD8a (1:200; Cell Signaling, 98941S), rabbit anti-phospho-histone H3 (Ser 10; 1:200; Cell Signaling, 9701S). Appropriate secondary antibodies were used, including Alexa 488-conjugated secondary antibodies (Invitrogen; 1:1,000) and biotin-labeled antibodies (Jackson Immunoresearch; 1:500–1:2,000), and, when necessary, TSA-fluorescein tyramide and TSA Plus Cyanine 3 System amplification (PerkinElmer, Life Sciences) were performed according to the manufacturer’s instructions. Hoechst 33342 (Invitrogen; 1:1,000) or hemaluin counterstaining were performed. DAB was added for visualization of CK19 staining (10 min). Apoptosis was detected by TUNEL assay kit (Sigma-Aldrich, 11684795910), according to the manufacturer’s instructions, followed by Hoechst 33342 (Invitrogen; 1:1,000) counterstaining. Slides were mounted with ProLong Gold mounting medium without DAPI (Invitrogen, P36930).

For detection of tumor hypoxia, tumors were collected 1 h after i.p. injection of 60 mg per kg (body weight) pimonidazole hydrochloride. To detect pimonidazole adducts, tumor sections were immunostained with Hypoxyprobe-1-Mab1 (Hypoxyprobe kit, Chemicon) following the manufacturer’s instructions. To analyze vessel perfusion, mice were retro-orbitally injected with 0.05 mg of FITC-conjugated lectin (*Lycopersicon esculentum*; Vector Laboratories). After 10 min, mice were perfused by intracardiac injection of saline for 5 min, and tumors were collected and immunostained as described. Imaging and microscopic analyses were performed with an Olympus BX41 microscope and CellSense imaging software.

### Lentiviral transductions

Cells were transduced with lentiviral vectors in medium containing 1 μg ml^–1^ polybrene. First, a vector containing Cas9 (under a doxycycline-inducible promoter) was used, followed by a transduction with a vector containing an sgRNA targeting the *Slc4a4* locus (5′-GATGAATCGGATGCGTTCTG-3′, first gRNA; 5′-GCCTCCAAAAGTGATGGCGT-3′, second gRNA) or a non-targeting control sgRNA (5′-GAACAGTCGCGTTTGCGACT-3′). A multiplicity of infection reaching approximately 30% of transduction was used. Transduced cells were selected with blasticidin (20 μg ml^–1^) and puromycin (2–5 μg ml^–1^), respectively. Cells were treated for 7 d with doxycycline (0.5 μg ml^–1^) to induce Cas9 expression and were kept in doxycycline-free medium for another 7 d before. Gene deletion was confirmed by western blotting.

*Ldha* and *Serpinb14* (ovalbumin) overexpression in cancer cells was driven under the control of a cytomegalovirus promoter. Control cells were transduced with empty vectors. Transduced cells expressing ovalbumin were selected with geneticin (Invivogen, G418). Cells transduced with the *Ldha* overexpression construct were sorted as CD90.1^+^ cells.

### Protein extraction and immunoblotting

Immunoblotting on whole-cell lysate was performed as previously described^64^. The following antibodies were used: rabbit anti-SLC4A4 (1:1,000; Abcam, ab187511), rabbit anti-LDHA (1:2,000; Novus Biologicals, NBP1-48336), mouse anti-CRISPR–Cas9 (1:1,000; Novus Biologicals, NBP2-36440V), rabbit anti-MCT4 (1:500; Proteintech, 22787-1-AP), rabbit anti-MCT1 (1:1,000; Proteintech, 20139-1-AP), anti-β-tubulin loading control HRP (1:2,000; Abcam, ab21058), mouse anti-vinculin (1:2,000; Sigma-Aldrich, V9131) and appropriate HRP-conjugated secondary antibodies (1:3,000; Cell Signaling, 7076S and 7074S). Signal was visualized by enhanced chemiluminescent reagents (ECL, Invitrogen) or West Femto (Thermo Scientific), according to the manufacturer’s instructions, and images were acquired by a LAS-4000-CCD camera with ImageQuant software (GE Healthcare).

### Radiolabeling assays

For bicarbonate uptake, cells were cultured for 2 min (37 °C) in M199 medium (Gibco; 10% FBS, 5 μCi ml^–1^ [^14^C]sodium bicarbonate) and lysed in 1 N NaOH. For glycolysis, cancer cells were incubated for 2 h in their culturing medium containing 0.4 μCi ml^–1^ [5-3H]-d-glucose (PerkinElmer, NET531001MC). Supernatant was transferred into glass vials sealed with rubber stoppers. ^3^H_2_O was captured on Whatman paper soaked in water for 48 h (37 °C). Radioactivity was determined by liquid scintillation counting.

### Microdialysis-based metabolite dosage

Glucose and lactate concentrations were measured using enzymatic assays (ISCUSflex Microdialysis Analyzer). Cells were seeded in 12-well plates and incubated for the indicated time points with medium (DMEM in powder (Sigma-Aldrich), pen/strep, sodium bicarbonate solution (7.5%; Sigma-Aldrich), dialyzed FBS and 10 mM glucose (Sigma-Aldrich)). The culture medium was collected in 10-kDa filtered tubes and analyzed by microdialysis according to manufacturer’s instructions.

### Seahorse experiment

ECAR and OCR were measured with the Seahorse-XF96 metabolic analyzer (Agilent). Cells (2 × 10^4^ per well) were seeded in 96-well plates. Glucose-dependent ECAR was assessed by calculating the difference of ECAR values before and after addition of 10 mM glucose in non-buffered DMEM (Sigma-Aldrich, D5030; pH 7.4, 2 mM glutamine). Basal OCR was assessed in non-buffered DMEM medium (pH 7.4, 2 mM glutamine and 10 mM glucose). Data were normalized to protein content.

### RNA extraction, reverse transcription and real-time quantitative PCR (RT–qPCR)

To extract RNA from tumors, samples were homogenized in 1 ml of TRIzol using a Ribolyser, followed by the addition of 200 μl of chlorofom, and centrifuged (10 min, 3,000*g*). RNA from cells and cDNA were obtained as previously described^63^. The cDNA, primer/probe mix and TaqMan Fast Universal PCR master mix were prepared according to manufacturer’s instructions (Applied Biosystems). Premade assays were purchased from IDT (*Slc4a4*, Mm.PT.58.30280518; *Lamp2*, Mm.PT.58.13168833; *Ldha*, Mm.PT.49a.8242615; *Mct1*, Mm.PT.58.7462799; *Hprt*, Mm.PT.58.32092191). For *Mct4* detection, forward (5′-TATCCAGATCTACCTCACCAC-3′) and reverse (5′-GGCCTGGCAAAGATGTCGATGA-3′) primers were used, and for *Gapdh* detection, forward (5′-GTGGAGTCATACTGGAACATGTAG-3′) and reverse (5′-AATGGTGAAGGTCGGTGTG-3′) primers were used; cDNA, primers and PowerUp SYBR Green master mix were prepared according to manufacturer’s instructions (Applied Biosystems).

### pH_i_ and pH_e_ measurements

Cells cultured on 12-mm coverslips, as described above, were incubated with 4 µM 2′,7′-bis-(2-carboxyethyl)-5-(and-6)-carboxyfluorescein-acetoxymethyl ester (BCECF-AM) for 1 h at room temperature (RT). The coverslip was placed in a perfusion cuvette. Using a Cary Eclipse spectrophotometer, cells were excited alternatively at 440 and 500 nm, and BCECF fluorescence emission was collected at 535 nm. The resting pH_i_ was measured in a Ringer solution with NaHCO_3_ at a pH_e_ of 7.4 (120 mM NaCl, 22 mM NaHCO_3_, 4.5 mM KCl, 1 mM CaCl_2_, 1 mM MgCl_2_ and 11 mM glucose), while Ringer KCl (20 mM NaCl, 110 mM KCl, 1 mM CaCl_2_, 1 mM MgSO_4_, 18 mM glucose and 20 mM HEPES) was adjusted at different pH values with KOH.

pH_i_ was estimated from the ratio of BCECF fluorescence calibrated by using the K^+^ nigericin method. The cells were incubated with 4 µM BCECF-AM and 5 µM nigericin in a KCl-rich medium for 1 h at RT. Subsequently, the cells were perfused with KCl medium at different pH values (6.7, 7, 7.4 and 8).

pH_e_ was measured directly in the cellular medium using single-barreled H^+^-sensitive microelectrodes, fabricated as described previously^65^ but with the following modifications. Briefly, single-barreled microelectrodes were constructed from a piece of filament-containing aluminum silicate glass tubing of 1.5-mm outer diameter and 1.0-mm inner diameter (Hilgenberg). Microelectrodes were pulled in a PE2 vertical puller (Narishige), silanized for 90 s in dimethyl-dichloro-silane vapor (Sigma) and baked in the oven for 3 h at 140 °C. The tip of the microelectrode was backfilled with proton ionophore cocktail (Hydrogen Ionophore II, Cocktail A; Sigma), and its shaft was later filled with a buffer solution (pH 7.0). The reference electrode was an Ag/AgCl wire connected to ground. All microelectrodes were calibrated before and after the measurements with NaCl solutions containing a mixture of KH_2_PO_4_ and Na_2_PO_4_ to yield pH values between 6.8 and 7.8. To measure the pH_e_ in close proximity of the cell membrane, the microelectrode was mounted on a Leitz micromanipulator and connected to a dual-channel electrometer (WPI) and a strip-chart recorder (Kipp and Zonen).

### Interstitial fluid collection

Intact tumors were collected into tubes with a perforated bottom and 20 µl of 0.9% NaCl solution (pH 7.4). Interstitial fluid was collected by centrifugation (110*g*, 10 min, 4 °C). Protein within the interstitial fluid was precipitated using −20 °C cold methanol/water mix (5:3) and centrifuged (20,000*g*, 5 min, 4 °C). The supernatant was analyzed by MS.

### Metabolite analysis by LC–MS/MS

To analyze metabolites from extracellular medium and tumor interstitial fluid, samples were collected and extracted with 80% methanol. Intracellular samples were centrifuged (20,000*g*, 10 min), and supernatant was used for analysis. Metabolite analysis by LC–MS/MS was performed as previously described^64^.

### Cell proliferation assay

Panc02 or KPC cells were seeded in 96-well plates, and cell growth was monitored with an S3 Incucyte for 100 h (optical module S3/SX1 G/R). Cell proliferation was calculated analyzing the occupied area of cells with the Incucyte Base analysis software.

### Apoptosis and cell cycle phase distribution

Cells and supernatant were collected in fluorescence-activated cell sorting (FACS) tubes. For apoptosis, samples were washed with Annexin V binding buffer (BioLegend, 422201) and stained in Annexin V binding buffer with Annexin antibody (1:25; BioLegend, 640941, APC) and propidium iodide (1:1,000; Sigma, P4864) for 15 min (RT). For cell cycle analysis, samples were washed and fixed in 70% ethanol, and DNA was extracted with a solution of Na_2_HPO_4_ (0.2 M) and citric acid (0.1 M; pH 7.8) for 10 min (37 °C). Subsequently, samples were washed and incubated for 30 min (37 °C) with 40 μg ml^–1^ propidium iodide (Sigma, P4864) and 100 μg ml^–1^ RNase. Sample analysis was performed with a FACS Fortessa (BD Biosciences). Data were analyzed by FlowJo (TreeStar).

### In vivo [^31^P]MRS and in vivo hyperpolarized 1-[^13^C] pyruvate MRS

MRS measurements were performed on Panc02 subcutaneous size-matched tumors on a dedicated 11.7 T small animal MRI (BioSpec, Bruker BioSpin). Animals were anesthetized by inhalation of isoflurane and warmed using a circulating water system. Respiration rate was monitored using a pressure cushion (SA Instruments).

For in vivo pH measurements, 3-aminopropyl phosphonate (3-APP; Sigma-Aldrich) was administered i.p. (11 mmol per kg (body weight)) 30 min before data acquisition. Experiments were performed using a ^1^H/^31^P-surface coil (2 cm in diameter, Bruker BioSpin) positioned over the tumor.

T2-weighted Rapid Acquisition with Relaxation Enhancement sequences in two different slice orientations were performed to select the tumor region. Localized ^31^P-NMR spectra were acquired using a pulse sequence with tumor volume selection based on outer volume suppression (bandwith of 10 kHz; *α*: 45°; average: 4,096; 2,048 points; repetition time : 500 ms; acquisition time: 34 min).

Using jMRUI v5, pH_i_ and pH_e_ measurements were calculated from the chemical shift between inorganic phosphate (P_i_) and α-ATP peaks and the 3-APP and α-ATP peaks, respectively, in the ^31^P spectra according to literature^66^.

[1‐^13^C]Pyruvate (Cortecnet) solution (40 μl) containing 15 mM trityl radical OX63 (GE Healthcare) and 2 mM gadolinium was hyperpolarized at 1.4 K and 3.35 T using an HyperSense DNP polarizer (Oxford Instruments). After 60 min, the solution was dissolved in 3 ml of a heated buffer containing 100 mg liter^–1^ EDTA, 40 mM HEPES, 30 mM NaCl, 80 mM NaOH and 30 mM non-hyperpolarized unlabeled lactate. Solution (250 μl) was administered intravenously to the mice, and ^13^C-spectra acquisition was started simultaneously.

Mice were scanned using a double tuned ^1^H/^13^C-surface coil (RAPID Biomedical) with a tumor-shaped cavity of 12 mm in diameter. Tumor volume was assessed with anatomic T2-weighted images. ^13^C-Spectra were acquired every 3 s for 210 s using a single-pulse sequence (bandwidth: 50 kHz; *α*: 10°; 10,000 points).

Peak areas under the curve were measured with MATLAB (Mathworks) for each repetition and time point. The integrated peak intensities of hyperpolarized [^13^C]pyruvate, [^13^C]lactate and total observed ^13^C-signal were used to calculate the lactate to pyruvate ratio.

### FACS analysis

Tumors were processed as previously described^67^ and stained with the following antibodies for surface markers: fixable viability dye (eFluor 506, 1:500), CD45 (30-F11, BUV395 or PerCP-Cy5.5, 1:200), TCRβ (H57-597, BV421 or FITC 1:300), CD4 (RM4-5, PerCP-Cy5.5 or BV711 1:500), CD8a (53-6.7, BUV805, 1:400), CD69 (H1.2F3, BUV605, 1:300), F4/80 (BM8, eFluor 450, 1:150), CD11b (M1/70, PE, 1:300), MHC class II (I-A/I-E M5/114.15.2, APC-Cy7, 1:500), CD206 (C068C2, Alexa Fluor 647, 1:100), CD11c (N418, PE-Cy7, 1:400), CD204 (REA148, FITC, 1:50), CD279/PD-1 (29F.1A12, BV421, 1:400) and CD274/PD-L1 (B7-H1, PE, 1:300), all from BD Biosciences.

For the intracellular measurement of IFNγ and GZMB, single-cell suspensions were cultured in RPMI (10% FBS and 1% pen/strep) and stimulated with phorbol 12-myristate 13-acetate/ionomycin cell stimulation cocktail (eBioscience, 1:500) in the presence of brefeldin A (BioLegend; 1:1,000) and monensin (eBioscience; 1:1,000) for 4 h (37 °C). Afterward, and for the intracellular measurement of Foxp3 and CTLA-4, cells were stained for surface markers (see before), followed by a 30-min incubation (4 °C) in Fix/Perm buffer (eBioscience, 00-5523). Cells were washed with permeabilization buffer (eBioscience, 00-5523) and stained overnight (4 °C) in permeabilization buffer with CTLA-4 (UC10-4B9, APC, 1:100), Foxp3 (FJK-16s, APC or PerCP-Cy5.5, 1:100), IFNγ (XMG1.2, PE-Cy7, 1:100) and GZMB (GB11, Alexa Fluor 647, 1:100). Cells were subsequently washed and resuspended in FACS buffer. FACS data were acquired using a FACS Fortessa (BD Biosciences). Data were analyzed by FlowJo (TreeStar).

### T cell isolation and activation

Naive mouse T cells were isolated from spleen by filtering the cells through a 40-μm pore cell strainer in sterile PBS. Red blood cell lysis buffer (Sigma-Aldrich) was used for red blood cell lysis. Total splenocytes were cultured in T cell medium (RPMI, 10% FBS, 1% pen/strep, 1% MEM non-essential amino acids, 25 μm β-mercaptoethanol (Gibco) and 1 mM sodium pyruvate (all Gibco)) at 37 °C in a humidified 5% CO_2_ incubator. T cells were activated for 3 d with CD3/CD28 Dynabeads (Thermo Fisher Scientific) at a 1:1 bead-to-cell ratio and 30 U ml^–1^ rIL-2 (PeproTech).

Total splenocytes from OT-I mice were isolated and cultured for 3 d in T cell medium with 1 µg ml^–1^ SIINFEKL peptide (IBA LifeSciences) and 30 U ml^–1^ rIL-2 (PeproTech).

### T cell cytotoxicity and proliferation assay

Green fluorescent protein (GFP)-labeled Panc02-OVA cancer cells (10 × 10^3^) were seeded in a 96-well plate. Activated OT-1 T cells were then added on top at a 1:5 target:effector ratio. Cells were cocultured in T cell medium alone or supplemented with 10 mM sodium lactate or 10 mM lactic acid or with the needed amount of HCl to reach pH 6.3 induced by the lactic acid condition. T cell killing was monitored with an Incucyte machine. For spheroid killing assays, 2 × 10^3^ GFP-labeled Panc02-OVA cancer cells were seeded in a 96-well U-bottom ultralow attachment plate (BRAND, 781900) in 100 µl per well. The plate was then centrifuged (10 min, 125*g*, RT), and spheroid formation was monitored. After 48 h, activated OT-1 T cells were added to the plate at a 1:5 target:effector ratio.

To measure proliferation, single-cell suspensions of whole splenocytes were cultured for 3 d in retronectin-coated (Takara) 48-well plates with CD3/CD28 Dynabeads (Thermo Fisher Scientific) in a medium composed of one-third T cell medium and two-thirds cancer cell conditioned medium or supplemented with an amount of lactic acid, HCl or sodium lactate resembling the lactate and pH level of sgNT control. pH levels were measured by pH meter, and lactate levels were measured with a colorimetric kit according to the manufacturer’s instructions (Spinreact). Maximal (+act) or basal (–act) CD8^+^ T cell proliferation was achieved by the presence or absence of CD3/CD28 antibodies in T cell medium. T cell killing and proliferation was monitored with an Incucyte machine.

### BMDM isolation and polarization

BMDMs were derived from BM precursors as previously described^64^. Briefly, BM cells isolated from C57BL/6 mice were cultured for 7 d in high-glucose DMEM (Gibco, 41965039; 20% FBS, 30% L929 conditioned medium, 25 mM HEPES, 2 mM l-glutamine and pen/strep). For the coculture experiments, BMDMs were added in a 1:3 ratio to Panc02 cancer cells in high-glucose DMEM (10% FBS and 1% pen/strep). After 24 h, cells were detached and processed for FACS staining. Macrophages were identified using the pan-macrophage-specific marker F4/80.

### Statistics and reproducibility

All statistical analyses were performed using GraphPad Prism software. Statistical significance was calculated by two-tailed paired or unpaired Student’s *t*-tests on two experimental conditions and one-way or two-way analysis of variance (ANOVA) test when more than two experimental groups were compared. For survival analysis, a log-rank (Mantel–Cox) test was used. All the results are shown as mean ± s.e.m.

No statistical method was used to predetermine sample size, but our sample sizes were selected based on those reported in previous studies^63,64,67^. Detection of mathematical outliers was performed using the Grubbs’ test in GraphPad. Animals were excluded only if they died or had to be killed according to protocols approved by the animal experimental committees. For in vitro experiments, no data were excluded. Data distribution was assumed to be normal, but this was not formally tested. The exact sample sizes are indicated in the figure legends. For in vivo studies, tumor measurement, treatment and analysis were performed blindly by different researchers to ensure that the studies were run in a blinded manner. Animals were randomized, with each group receiving mice with similar tumor size or similar body weight. For in vitro studies, randomization and blinding of cell lines was not possible; however, all cell lines were treated identically without prior designation. For in vitro experiments, at least two to three biological replicates were performed with similar results. For in vivo studies, at least five animals were allocated per group. Further information on research design is available in the Nature Research Reporting Summary linked to this article.

### Reporting summary

Further information on research design is available in the Nature Portfolio Reporting Summary linked to this article.

## Supplementary information

Reporting Summary

## Data Availability

Single-cell RNA-seq data that support the findings of this study have been deposited in the European Genome–Phenome Archive (EGA) under study number EGAS00001006334 and with data accession number EGAD00001008961. Requests for accessing raw sequencing data will be reviewed by the UZ Leuven-VIB Data Access Committee (dac@vib.be). Any data shared will be released via a Data Transfer Agreement that will include the necessary conditions to guarantee protection of personal data (according to European GDPR law). Single-cell RNA-seq data from the second cohort of human PDAC samples can be found in ref. ^21^ with accession number GSA CRA001160. The bulk RNA-seq human PDAC data were derived from the TCGA Research Network. TGCA data were downloaded from the UCSC Xena platform (http://xena.ucsc.edu/). Source data are provided with this paper. All other data supporting the findings of this study are available from the corresponding author on reasonable request.

## Source data

Source Data Fig. 1 Statistical source data.
Source Data Fig. 2 Statistical source data.
Source Data Fig. 3 Statistical source data.
Source Data Fig. 4 Statistical source data.
Source Data Fig. 5 Statistical source data.
Source Data Fig. 6 Statistical source data.
Source Data Fig. 7 Statistical source data.
Source Data Fig. 7 Unprocessed western blot.
Source Data Fig. 8 Statistical source data.
Source Data Extended Data Fig. 1 Statistical source data.
Source Data Extended Data Fig. 2 Statistical source data.
Source Data Extended Data Fig. 2 Unprocessed western blot.
Source Data Extended Data Fig. 3 Statistical source data.
Source Data Extended Data Fig. 3 Unprocessed western blot.
Source Data Extended Data Fig. 4 Statistical source data.
Source Data Extended Data Fig. 5 Statistical source data.
Source Data Extended Data Fig. 6 Statistical source data.
Source Data Extended Data Fig. 7 Statistical source data.
