# Supplementary material for: Targeting the bicarbonate transporter SLC4A4 overcomes immunosuppression and immunotherapy resistance in pancreatic cancer
Source: Nat Cancer. 2022 Dec 15;3(12):1464–83. doi: 10.1038/s43018-022-00470-2 (PMC9767871; doi:10.1038/s43018-022-00470-2)
Supplement: Supplementary file 1 — Reporting Summary [file 43018_2022_470_MOESM1_ESM.pdf]

nature portfolio

Corresponding author(s): Massimiliano Mazzone

Last updated by author(s): Oct 18, 2022

Reporting Summary

Nature Portfolio wishes to improve the reproducibility of the work that we publish. This form provides structure for consistency and transparency in reporting. For further information on Nature Portfolio policies, see our Editorial Policies and the Editorial Policy Checklist.

Statistics

For all statistical analyses, confirm that the following items are present in the figure legend, table legend, main text, or Methods section.

n/a	Confirmed	
☐	☒	The exact sample size (*n*) for each experimental group/condition, given as a discrete number and unit of measurement
☐	☒	A statement on whether measurements were taken from distinct samples or whether the same sample was measured repeatedly
☐	☒	The statistical test(s) used AND whether they are one- or two-sided<br>*Only common tests should be described solely by name; describe more complex techniques in the Methods section.*
☐	☒	A description of all covariates tested
☐	☒	A description of any assumptions or corrections, such as tests of normality and adjustment for multiple comparisons
☐	☒	A full description of the statistical parameters including central tendency (e.g. means) or other basic estimates (e.g. regression coefficient) AND variation (e.g. standard deviation) or associated estimates of uncertainty (e.g. confidence intervals)
☐	☒	For null hypothesis testing, the test statistic (e.g. *F*, *t*, *r*) with confidence intervals, effect sizes, degrees of freedom and *P* value noted<br>*Give P values as exact values whenever suitable.*
☒	☐	For Bayesian analysis, information on the choice of priors and Markov chain Monte Carlo settings
☒	☐	For hierarchical and complex designs, identification of the appropriate level for tests and full reporting of outcomes
☒	☐	Estimates of effect sizes (e.g. Cohen's *d*, Pearson's *r*), indicating how they were calculated

Our web collection on statistics for biologists contains articles on many of the points above.

Software and code

Policy information about availability of computer code

Data collection	CellSense imaging software (v1.18) was used for imaging data acquisition. QuantStudio TM 12K Flex software (v1.4) was used for real-time PCR. ImageQuant software (v1.2) was use for immunoblot images acquisition. Incucyte Base analysis (v2018A) software was used for incucyte imaging collection. Single-cell sequencing samples were processed with the Cellranger (v3.1.0). Flow cytometry data collection was done with BD FACs DIVA software (v9.0)
Data analysis	All statistical analyses were performed using GraphPad Prism software (v9.4.1). Single-cell sequencing data was analyzed with the Scanpy software (v1.6.0). The ggplot2 package of programming language R (v4.0.4) was used to create violin plots. Flow cytometry analysis was done with the FlowJo software (v10.8.1). Incucyte Base analysis (v2018A) software was used for incucyte imaging analysis. ImageJ (v1.53) was used for Western Blot image analysis.

For manuscripts utilizing custom algorithms or software that are central to the research but not yet described in published literature, software must be made available to editors and reviewers. We strongly encourage code deposition in a community repository (e.g. GitHub). See the Nature Portfolio guidelines for submitting code & software for further information.

Data

Policy information about availability of data

All manuscripts must include a data availability statement. This statement should provide the following information, where applicable:

- Accession codes, unique identifiers, or web links for publicly available datasets
- A description of any restrictions on data availability
- For clinical datasets or third party data, please ensure that the statement adheres to our policy

Single cell RNA–seq data that support the findings of this study have been deposited in the European Genome-phenome Archive (EGA) under study no. EGAS00001006334 and with data accession no. EGAD00001008961. Requests for accessing raw sequencing data will be reviewed by the UZ Leuven-VIB Data Access Committee (dac@vib.be). Any data shared will be released via a Data Transfer Agreement that will include the necessary conditions to guarantee protection of personal data (according to European GDPR law). Single-cell RNA-seq data from the second cohort of PDAC patients can be found in Peng, J. et al23 with accession number GSA: CRA001160. The bulk RNA-seq human PDAC data were derived from the TCGA Research Network. TGCA data was downloaded from the UCSC Xena platform (http://xena.ucsc.edu/).
Source data for Fig. 1-8 and Extended Data Fig. 1-7 have been provided as Source Data files. All other data supporting the findings of this study are available from the corresponding author on reasonable request.

Human research participants

Policy information about studies involving human research participants and Sex and Gender in Research.

Reporting on sex and gender	As outlined in the method, for RNA-seq analysis we collected 3 PDAC samples from male patients and 7 samples from female patients, while for IHC we collected 5 males and 2 females samples. No gender related issues are applied to this analysis.
Population characteristics	For RNA-seq human PDAC samples were obtained from 10 treatment-naïve patients (7 females, 3 males) median age 66,5 (range 47-81 years). For IHC samples were obtained from 7 patients (2 females, 5 males) median age 64 (range 42-73 years).
Recruitment	Resection material was collected from primary tumors during surgery. The presence of adenocarcinoma was proven on histopathology.
Ethics oversight	The study was approved by the Ethical Committee of the University Hospitals KU Leuven (Leuven, Belgium) with the reference number ML3452 and patients were given informed consent

Note that full information on the approval of the study protocol must also be provided in the manuscript.

Field-specific reporting

Please select the one below that is the best fit for your research. If you are not sure, read the appropriate sections before making your selection.

☒ Life sciences ☐ Behavioural & social sciences ☐ Ecological, evolutionary & environmental sciences

For a reference copy of the document with all sections, see nature.com/documents/nr-reporting-summary-flat.pdf

Life sciences study design

All studies must disclose on these points even when the disclosure is negative.

Sample size	No statistical method was used to predetermine sample size, but our sample sizes were selected based on those reported in previous studies (Celus et al. Cancer Immunol. Res. 2022; Virga et al. Sci. Adv. 2021; Bieniasz-Krzywiec et al. Cell Met 2019)
Data exclusions	Detection of mathematical outliers was performed using the Grubbs' test in GraphPad. Animals were excluded only if they died or had to be killed according to protocols approved by the animal experimental committees. For in vitro experiments no data were excluded.
Replication	For in vitro experiments at least two-three biological replicates were performed with similar results. For in vivo studies at least 5 animals were allocated per group.
Randomization	Animals were randomized with each group receiving mice with similar tumor size or similar body weight.
Blinding	For in vivo studies, the tumor measurement, treatment and analysis were performed blindly by different researchers to ensure that the studies were run in a blinded manner. For in vitro studies, randomization and blinding of cell lines was not possible; however, all cell lines were treated identically without prior designation.

Reporting for specific materials, systems and methods

We require information from authors about some types of materials, experimental systems and methods used in many studies. Here, indicate whether each material, system or method listed is relevant to your study. If you are not sure if a list item applies to your research, read the appropriate section before selecting a response.

Materials & experimental systems

n/a	Involved in the study
☐	☒ Antibodies
☐	☒ Eukaryotic cell lines
☒	☐ Palaeontology and archaeology
☐	☒ Animals and other organisms
☒	☐ Clinical data
☒	☐ Dual use research of concern

Methods

n/a	Involved in the study
☒	☐ ChIP-seq
☐	☒ Flow cytometry
☒	☐ MRI-based neuroimaging

Antibodies

Antibodies used

For western blot: Slc4a4 (Abcam, ab187511, polyclonal, 1:1000), Ldha (Novus biologicals, NBP1-48336, polyclonal, 1:2000), Cas9 (Novus biologicals, NBP2-36440V, 7A9-3A3, 1:1000), MCT4 (Proteintech, 22787-1-AP, polyclonal, 1:500), MCT1 (Proteintech, 20139-1-AP, polyclonal, 1:1000), Vinculin (Sigma-Aldrich, V9131, hVIN-1, 1:2000), Beta-tubulin (Abcam, ab21058, polyclonal, 1:2000), HRP-conjugated secondary antibodies (Cell Signalling, anti-mouse; 7076S, anti-rabbit; 7074S, 1:3000)
For histology : Rabbit anti-SLC4A4 (Abcam, ab187511, polyclonal, 1:5000), Rat anti-F4/80 (Serotec, MCA497F, Cl:A3-1, 1:100), Rat anti-CD34 (BD Pharmigen, 553731, RAM34, 1:100), Mouse anti MMR/CD206 (R&D system, AF2535, polyclonal, 1:100), Rabbit anti-FITC (Serotec, 4510-7604, polyclonal, 1:200), Rabit anti-CK19 (Abcam, ab15463, polyclonal, 1:100), Rabbit anti-CD8a (Cell signalling, 98941S, D4W2Z, 1:200), Rabbit anti phospho-histone-3 (Ser10) (Cell signalling, 9701S, polyclonal, 1:200)
For FACs: Fixable viability dye (eFluor™ 506, BD Bioscience, 65-0866-18, 1:500),CD45 (BUV395 or PerCP-Cy5.5, BD Bioscience, 564279 or 103132 ,30-F11, 1:200), TCRb (BV421 or FITC, BD Bioscience, 562839 or 553170, H57-597, 1:300), CD4 (PerCP-Cy5.5 or BV711, BD Bioscience, 100540, RM4-5, 1:500), CD8a (BUV805, BD Bioscience, 612898, 53-6.7, 1:400), CD69 (BUV605, BD Bioscience, 104529, H1.2F3, 1:300), F4/80 (eFluor™ 450, eBioscience, 48-4801-80, BM8, 1:150), CD11b (PE, BD Bioscience, 557397, M1/70, 1:300), MHC-II (APC-Cy7, Sony Biotechnology, RT1138140, I-A/I-E M5/114.15, 1:500), CD206 (Alexa Fluor 647, Biolegend, 141712 C068C2, 1:100), CD11c (PE-Cy7, eBioscience, 25-0114-82,N418, 1:400), CD204 (FITC, Miltenyi Biotec, 130-102-251, REA148, 1:50), CD279/PD-1 (BV421, Biolegend, 135221, 29F.1A12, 1:400) and CD274/PD-L1 (PE, Biolegend, 124307, B7-H1, 1:300), CTLA-4 (APC, Biolgend, 106309, UC10-4B9, 1:100), Foxp3 (APC or PerCP-Cy5.5, eBioscience, 17-5773-82 or 45-5773-82, FJK-16s, 1:100), IFNg (PE-cy7, eBioscience, 25-7311-82, XMG1.2, 1:100) and GZMB (Alexa Fluor 647, Biolgend, 515406, GB11, 1:100)
For in vivo experiments: Rat serum IgG (Sigma-Aldrich, I4131, 10mg/kg); Ultra-LEAF™ Purified PD-1anti-mouse (CD279) (BioLegend, 96167, RMP1-14, 10mg/kg); InVivoMAb anti-mouse CTLA-4 (CD152) (BioCell, BE0164, 9D9, 10mg/kg); InVivoMAb anti-mouse CD8α (BioCell, BE0004-1, 53-6.7, 10mg/kg).

Validation

All antibodies have been validated for use in their respective application as stated in the manufacturer's product pages.
For western blot:
Slc4a4 (Abcam, ab187511, polyclonal) suitable for WB and IHC. Reacts with Human, mouse and rat. Slc4a4-KO cells were used as negative control.
Ldha (Novus biologicals, NBP1-48336, polyclonal), suitable for WB. Reacts with Human,mouse, porcine and bovine. Ldha-overexpressing cells were use as positive control.
Cas9 (Novus biologicals, NBP2-36440V, 7A9-3A3), suitable for WB. Reacts with mouse. Cas9 constitutive expressing cells were used as positive control.
MCT4 (Proteintech, 22787-1-AP, polyclonal), suitable for WB. Reacts with Human, mouse and rat. MCT4-KO cells were used as negative control.
MCT1 (Proteintech, 20139-1-AP, polyclonal), suitable for WB. Reacts with Human, mouse, rat and bovine. MCT1-KO cells were used as negative control.
Vinculin (Sigma-Aldrich, V9131, hVIN-1), suitable for WB. Reacts with bovine, canine, mouse, rat, turkey, human, chicken, frog.
Beta-tubulin (Abcam, ab21058, polyclonal), suitable for WB. Reacts with Human, mouse and rat.
For histology and immunostainings: As negative control, one section per slide was stained according to the same protocol omitting the primary antibody.
Rabbit anti-SLC4A4 (Abcam, ab187511, polyclonal), suitable for WB and IHC. Reacts with Human, mouse and rat.
Rat anti-F4/80 (Serotec, MCA497F, Cl:A3-1), suitable for IF. Reacts with mouse.
Rat anti-CD34 (BD Pharmigen, 553731, RAM34), suitable for IF. Reacts with mouse.
Mouse anti MMR/CD206 (R&D system, AF2535, polyclonal), suitable for IF. Reacts with mouse.
Rabit anti-CK19 (Abcam, ab15463, polyclonal), suitable for IHC. Reacts with human and predicted to react with mouse.
Rabbit anti-CD8a (Cell signalling, 98941S, D4W2Z), suitable for IF. Reacts with mouse.
Rabbit anti phospho-histone-3 (Ser10) (Cell signalling, 9701S, polyclonal) suitable IF. Reacts with Human, mouse and rat.
For FACS analysis: FMO (fluorescence minus one) was evaluated for every antibody to assess specificity in FACS stainings.

Eukaryotic cell lines

Policy information about cell lines and Sex and Gender in Research

Cell line source(s)	Panc02 cells were kindly provided by Prof. B. Wiedenmann (Charité, Berlin) and originally purchased from ATCC. The 3 murine pancreatic ductal adenocarcinoma KPC cell lines were kindly provided by Hanahan's lab at the École Polytechnique Fédérale de Lausanne (EPFL) and they were generated from FVB mice carrying different genetic mutations P48Cre/KrasG12D/p53LSL R172H.
Authentication	Cell lines were authenticated based on morphological criteria. Once thawed, cells were not kept for longer than 10 passages in a humidified incubator in 5% CO2 and 95% air at 37 ºC. An internal golden stock of all cell lines was generated and maintained by the Lab Manager.
Mycoplasma contamination	All cell lines were confirmed to be mycoplasma-free by PlasmoTest™ - Mycoplasma Detection Kit (InvivoGen).
Commonly misidentified lines (See ICLAC register)	No commonly misidentified cell lines were used.

Animals and other research organisms

Policy information about studies involving animals; ARRIVE guidelines recommended for reporting animal research, and Sex and Gender in Research

Laboratory animals	FBV, C57BL6/N and NMRI nu/nu athymic nude mice were purchased from Envigo. Rag2/OT-1 mice were purshased from Taconic. Akk mice used for tumor experiments were females between 8 and 12 weeks old. Mice were maintained under pathogen-free, temperature- and humidity-controlled conditions under a 12/12-h light/dark cycle and received normal chow (ssniff® R/M-H).
Wild animals	No wild animals were used for this study.
Reporting on sex	The phenotypes were observed indiscriminately in male and female mice. No gender related issues are applied to this work.
Field-collected samples	No filed-collected samples were used for this study
Ethics oversight	Housing and all experimental animal procedures were approved by the Institutional Animal Care and Research Advisory Committee of the KU Leuven (P226/2017). Animals were removed from the study and killed if any signs of pain and distress were detected or if the tumor volume reached 2000mm3. The maximal tumor size was not exceeded in all reported studies.

Note that full information on the approval of the study protocol must also be provided in the manuscript.

Flow Cytometry

Plots

Confirm that:

☒ The axis labels state the marker and fluorochrome used (e.g. CD4-FITC).

☒ The axis scales are clearly visible. Include numbers along axes only for bottom left plot of group (a 'group' is an analysis of identical markers).

☒ All plots are contour plots with outliers or pseudocolor plots.

☒ A numerical value for number of cells or percentage (with statistics) is provided.

Methodology

Sample preparation	Mice were sacrificed by cervical dislocation and the tumors were harvested in cold PBS. Tumors were minced in alpha MEM (Lonza) containing 0,085 mg/ml Collagenase V (Sigma), 0,125 mg/ml Collagenase D (Roche), 0,1 mg/ml Dispase (Gibco), 5U/ml DNase I (Sigma) and 50 µM mercaptoethanol (Gibco) and incubated in the same solution for 30 minutes at 37°C. The digested tissues were filtered using a 70-µm pore sized strainer and cells were centrifuged 5 minutes at 300 xg. Red blood cell lysis was performed by using a home-made red blood cell lysis buffer (150 mM NH4Cl, 0.1 mM EDTA, 10 mM KHCO3, pH 7.4)
Instrument	FACS LRSFortessa X-20 (BD Bioscience, model number 658226R1)
Software	Flow cytometry data collection was done with BD FACs DIVA software (v9.0)<br>Flow cytometry analysis was done with the FlowJo software (v10.8.1).
Cell population abundance	This study did not involve sorting.
Gating strategy	Macrophages were gated for FSC/SSC, CD45 positive/alive, F4/80 and CD11b positive. MHC-II positive and CD206 positive were gated out of macrophages.

T cells were gated for FSC/SSC, CD45 positive/alive and TCRb positive. Tregs were gated for FSC/SSC, CD45 positive/alive, TCRb positive, CD4 positive and Foxp3 positive. CD8+ T cells were gated for FSC/SSC, CD45 positive/alive, TCRb positive and CD8 positive. IFNgamma positive cells were gated out of CD8+ T cells.
CD45 negative cells were gated for FSC/SSC, alive and CD45 negative.

☒ Tick this box to confirm that a figure exemplifying the gating strategy is provided in the Supplementary Information.
