# Supplementary figures and images for: Targeting the bicarbonate transporter SLC4A4 overcomes immunosuppression and immunotherapy resistance in pancreatic cancer
Source: Nat Cancer. 2022 Dec 15;3(12):1464–83. doi: 10.1038/s43018-022-00470-2 (PMC9767871; doi:10.1038/s43018-022-00470-2)

LDHA expression (KPC#1)

Fig. 7a

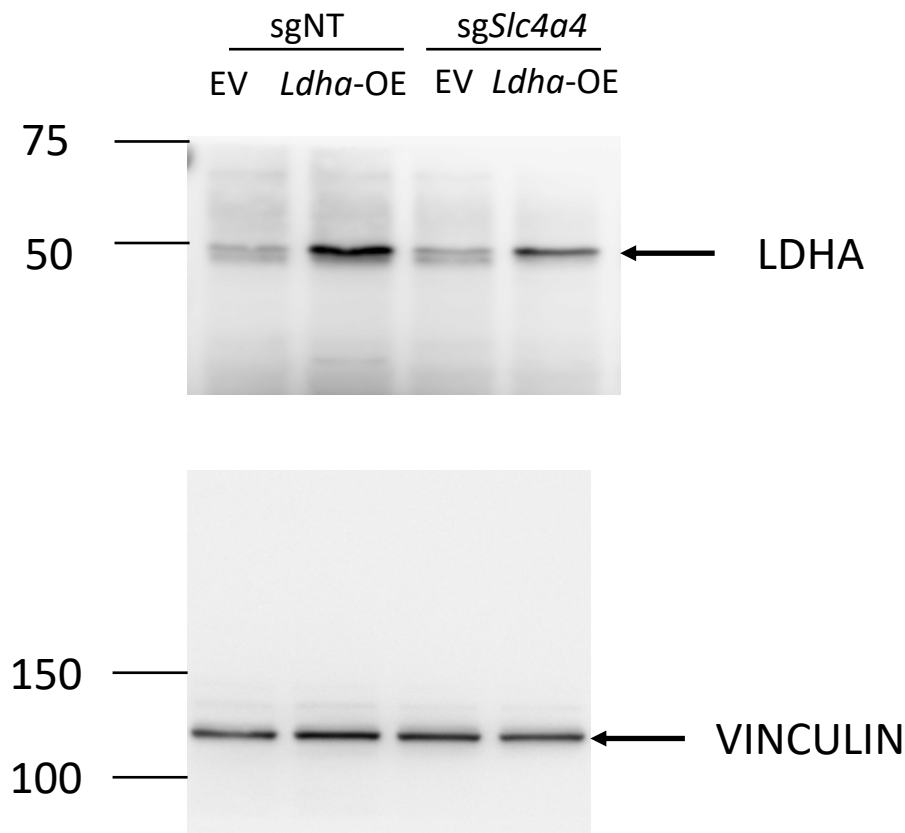

Supplement: Source Data Fig. 7 — Unprocessed western blot. [file 43018_2022_470_MOESM9_ESM.pdf]
