# Supplementary material for: Targeting the bicarbonate transporter SLC4A4 overcomes immunosuppression and immunotherapy resistance in pancreatic cancer
Source: Nat Cancer. 2022 Dec 15;3(12):1464–83. doi: 10.1038/s43018-022-00470-2 (PMC9767871; doi:10.1038/s43018-022-00470-2)

SLC4A4 expression (Panc02)

Extended Data Fig. 2a

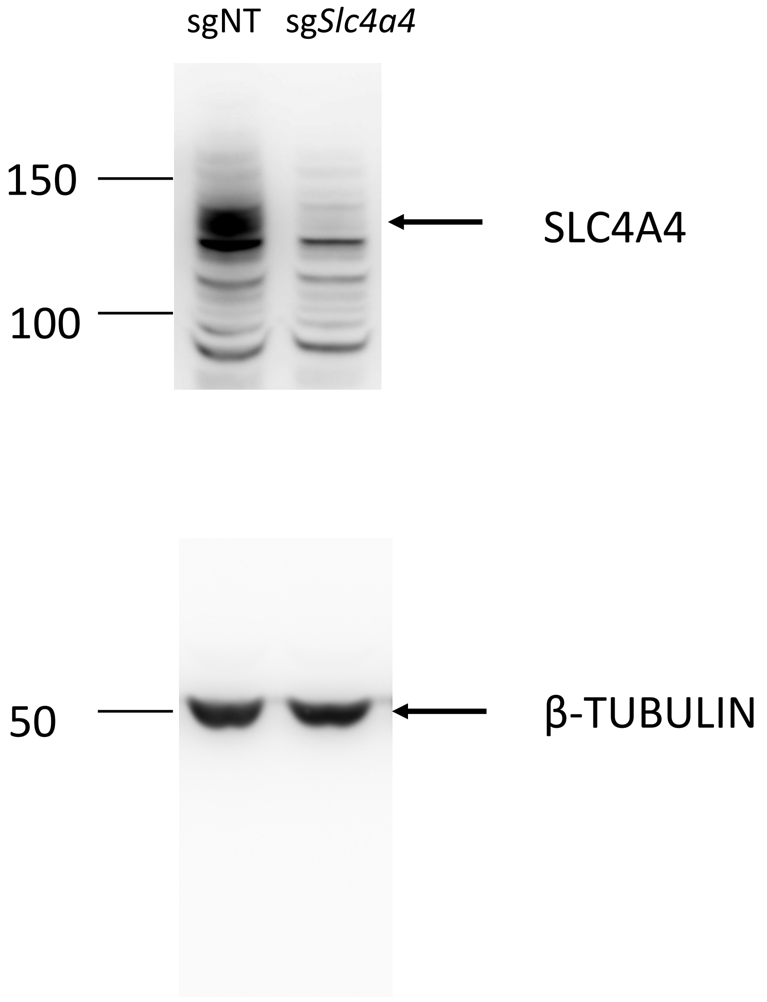

SLC4A4 expression (KPC#1)

Extended Data Fig. 2b

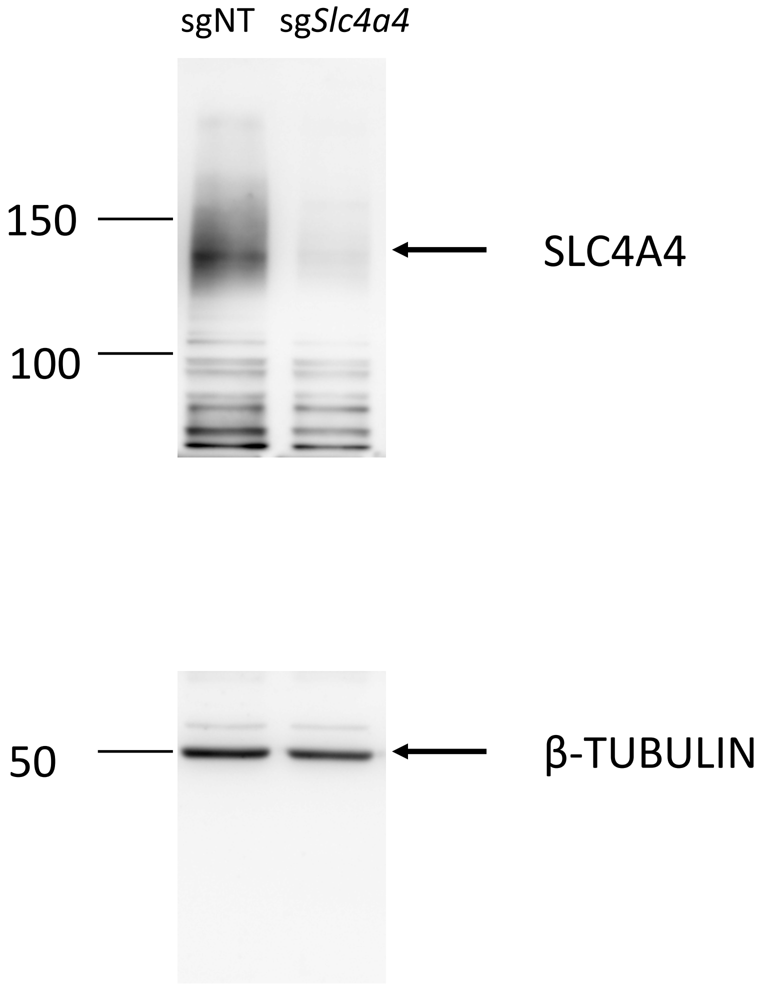

SLC4A4 expression (KPC#2)

Extended Data Fig. 2c

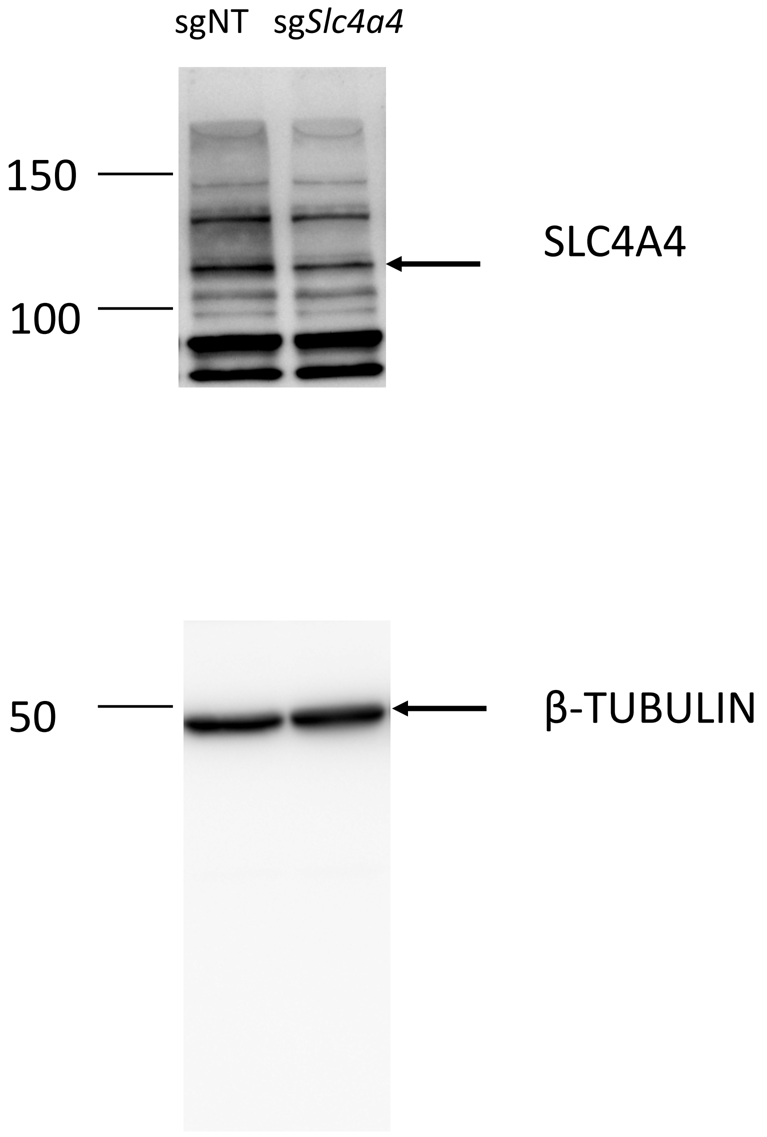

CAS9 expression

Extended Data Fig. 2d

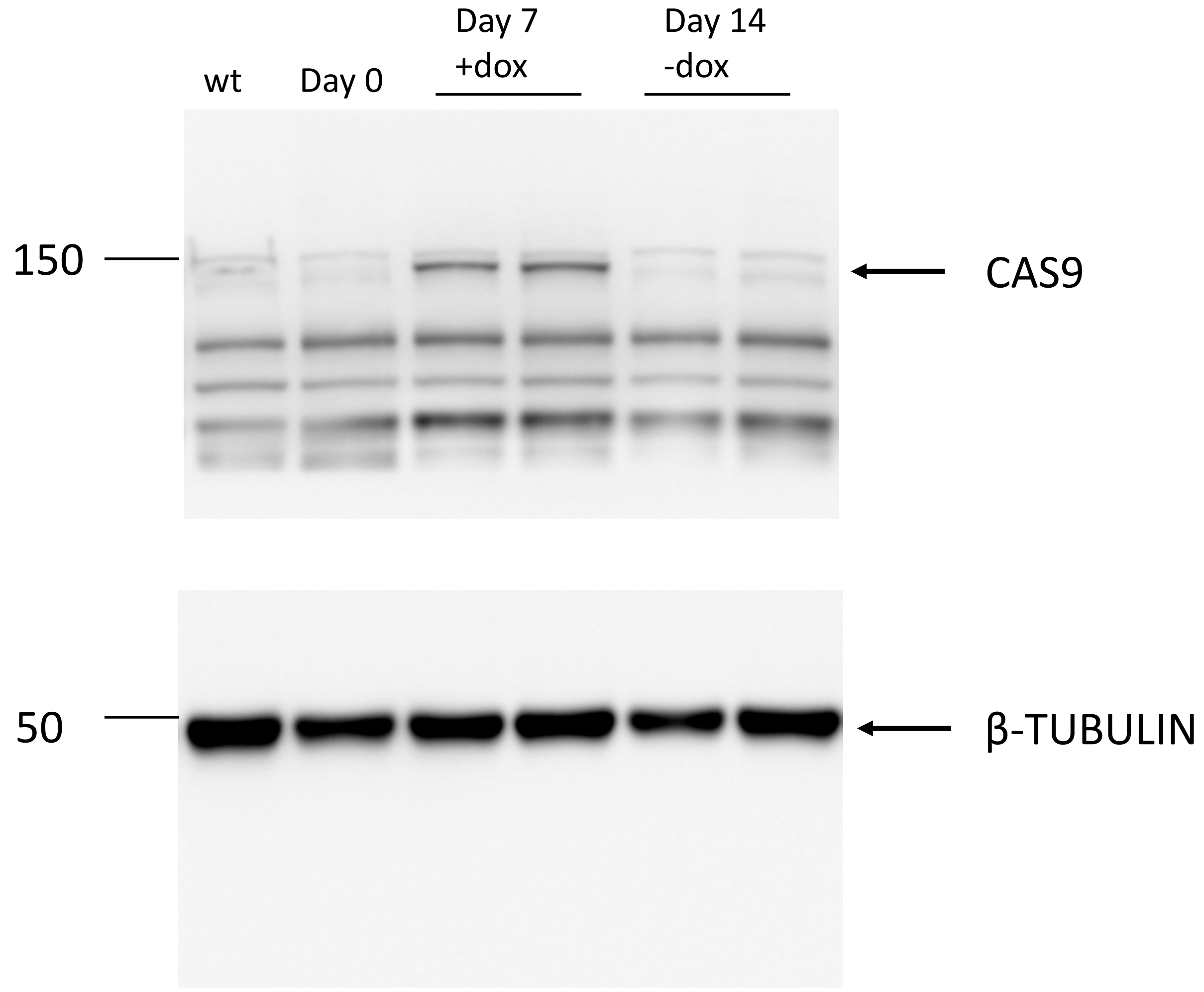

SLC4A4 expression (Panc02 2nd gRNA)

Extended Data Fig. 2k

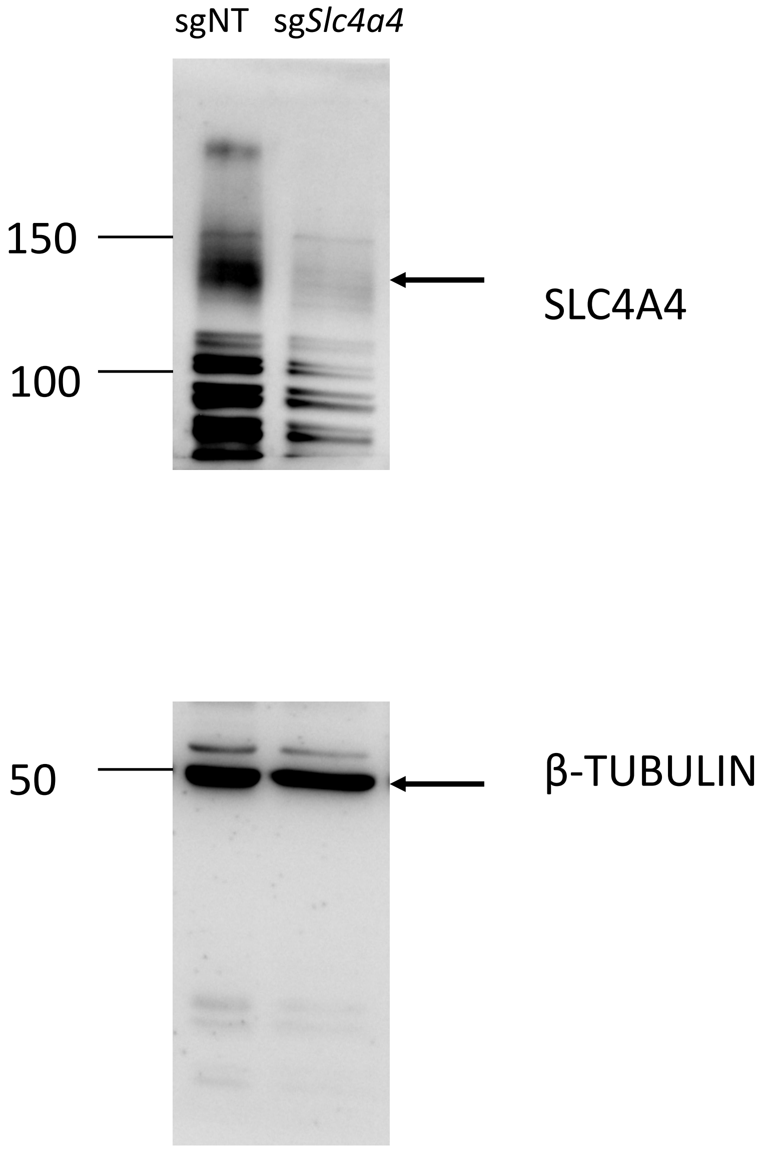

SLC4A4 expression (KPC#3)

Extended Data Fig. 2n

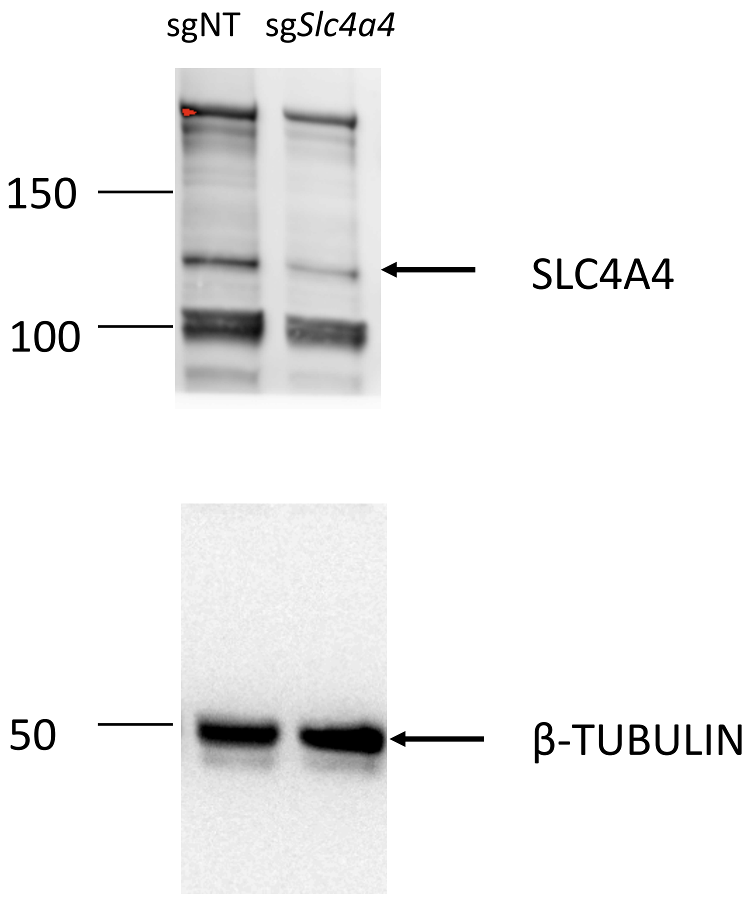

Supplement: Source Data Extended Data Fig. 2 — Unprocessed western blot. [file 43018_2022_470_MOESM13_ESM.pdf]
