# Supplementary material for: Targeting the bicarbonate transporter SLC4A4 overcomes immunosuppression and immunotherapy resistance in pancreatic cancer
Source: Nat Cancer. 2022 Dec 15;3(12):1464–83. doi: 10.1038/s43018-022-00470-2 (PMC9767871; doi:10.1038/s43018-022-00470-2)

LDHA expression (Panc02)

Extended Data Fig. 3e

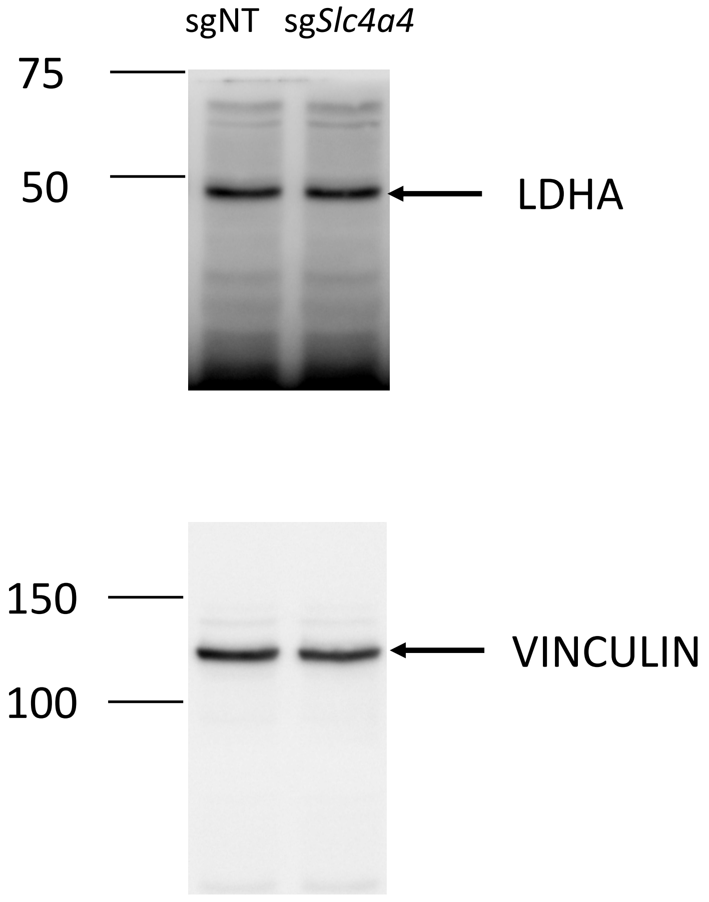

LDHA expression (KPC#1)

Extended Data Fig. 3f

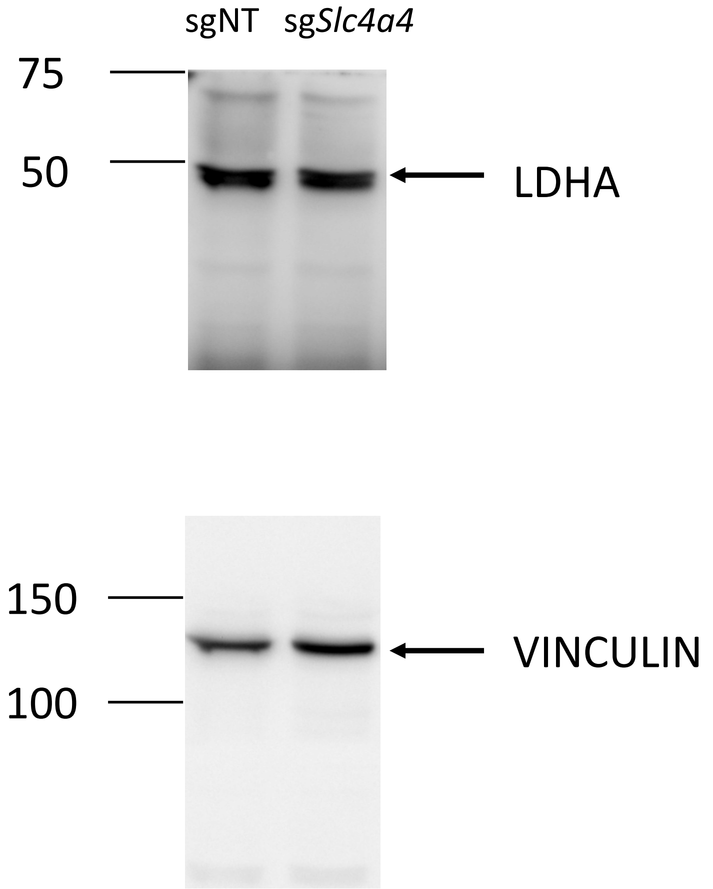

MCT1 expression (Panc02)

Extended Data Fig. 3g

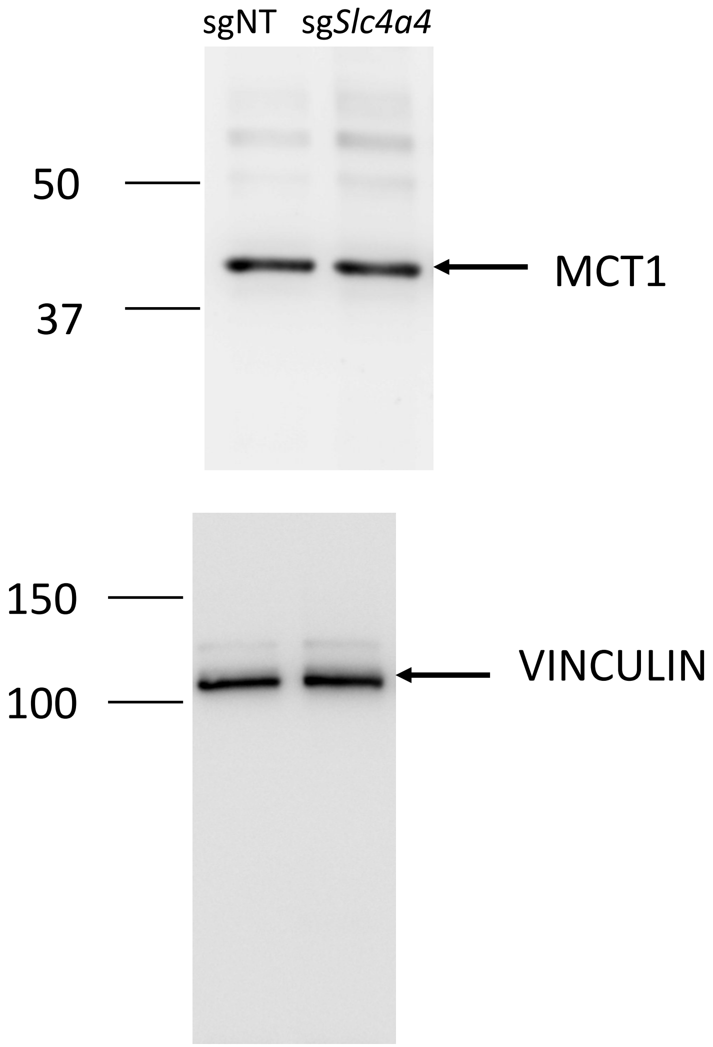

MCT1 expression (KPC#1)

Extended Data Fig. 3h

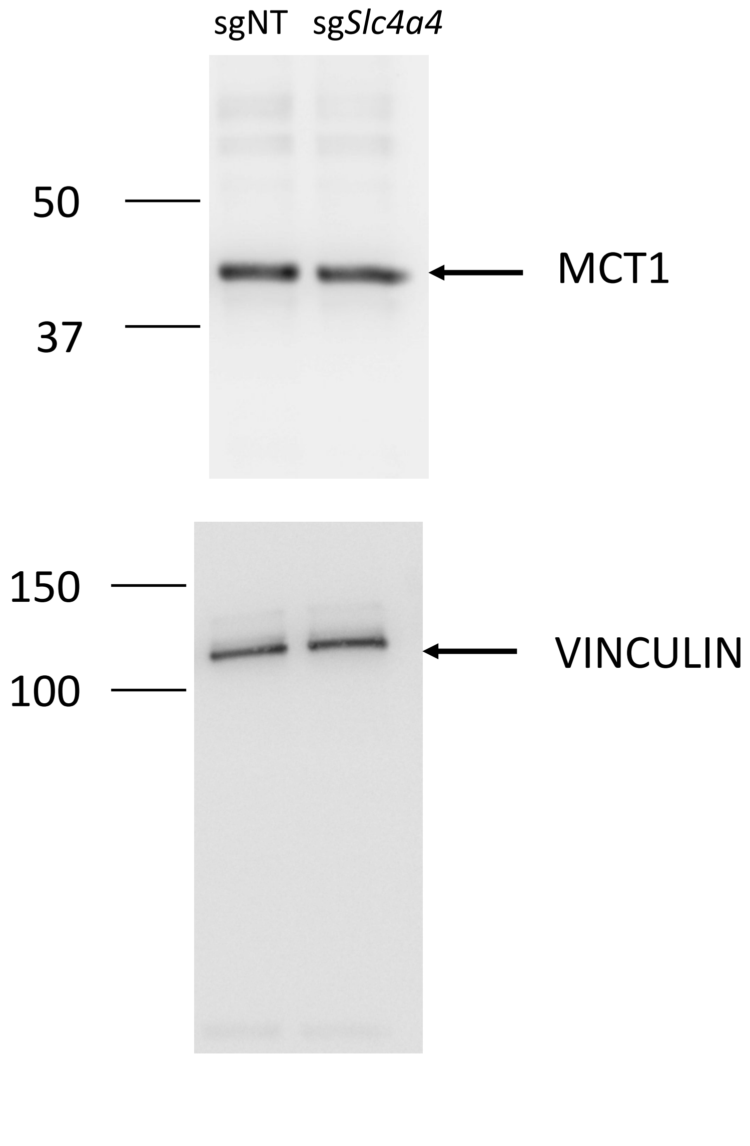

MCT4 expression (Panc02)

Extended Data Fig. 3i

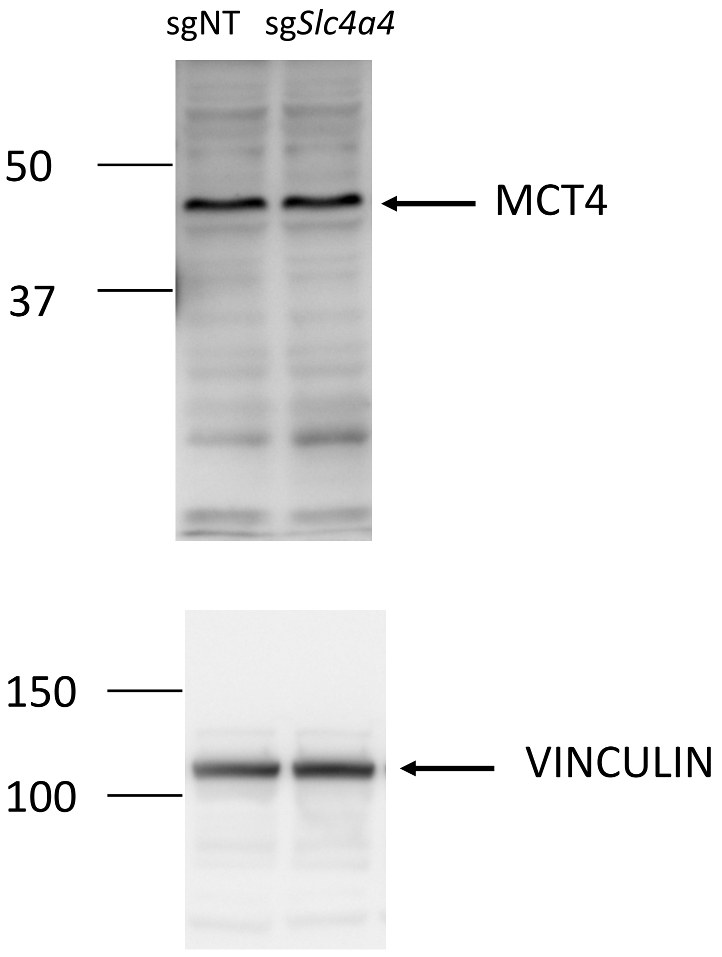

MCT4 expression (KPC#1)

Extended Data Fig. 3j

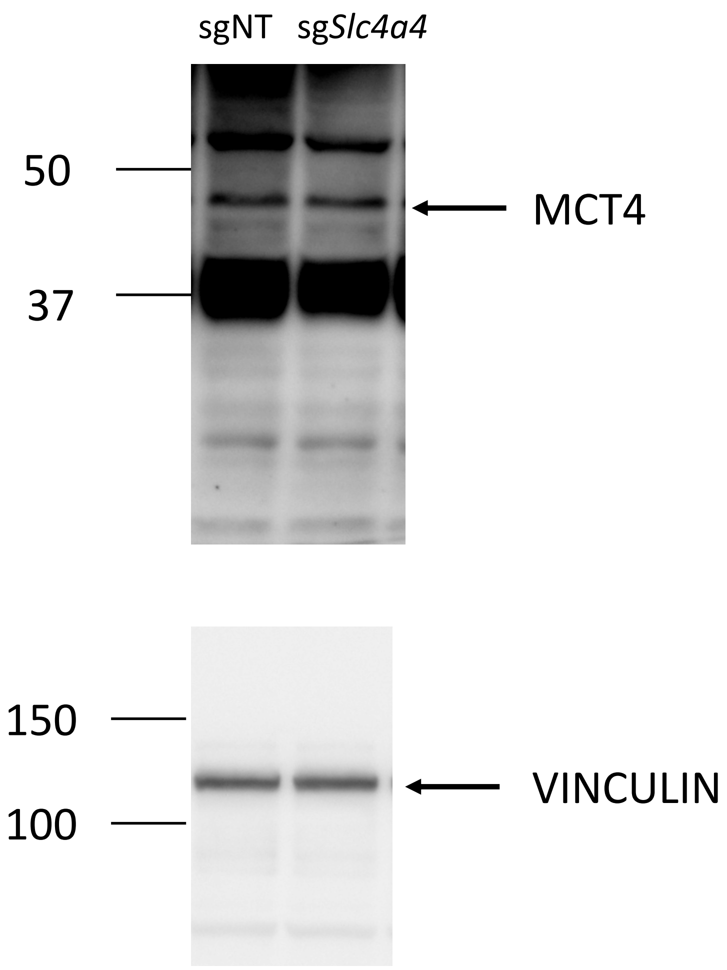

Supplement: Source Data Extended Data Fig. 3 — Unprocessed western blot. [file 43018_2022_470_MOESM15_ESM.pdf]
